# Design, synthesis and biological evaluation of a β-galactosidase-activated glycopeptide analogue of somatostatin

**DOI:** 10.1039/d5ra08677a

**Published:** 2026-07-21

**Authors:** Connor J. O'Leary, Sara Corbezzolo, Ailish Newton, Alby Benny, Eoin M. Scanlan, Krystian Kolodziejczak, Michael G. Monaghan, Joanna F. McGouran

**Affiliations:** a Trinity Biomedical Sciences Institute, School of Chemistry, Trinity College Dublin 152-160 Pearse St D02 R590 Ireland jmcgoura@tcd.ie; b SSPC, the Research Ireland Centre for Pharmaceuticals Ireland; c Discipline of Mechanical, Manufacturing & Biomedical Engineering, Trinity College Dublin Dublin 2 Ireland; d Ringaskiddy Active Pharmaceutical Ingredient Plant, Pfizer Ireland Pharmaceuticals Ringaskiddy, Co. Cork Ireland

## Abstract

Here we report a glycopeptide analogue of somatostatin, selectively activated by β-galactosidase. A solid-phase peptide synthesis compatible glycoamino acid masked the peptide pharmacophore, blocking receptor binding. β-Galactosidase activation in cancer cells enhanced cytotoxicity, demonstrating a model system for targeted peptide delivery and recognition with enzyme-mediated spatiotemporal control.

## Abstract 2

Here we report a glycopeptide analogue of somatostatin, selectively activated by β-galactosidase to mediate an increase in cell cytotoxicity.

## Graphical abstract

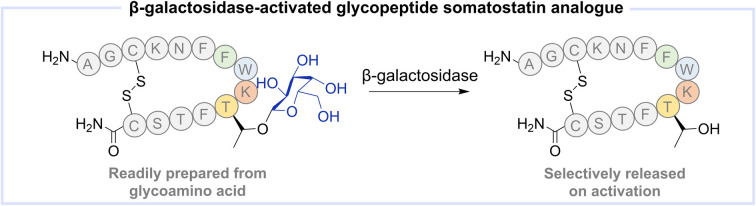

Many anticancer agents, have limited pre-clinical and clinical success due to dose-limiting toxicity arising from their unsatisfactory specificity for cancer cells.^1^ Rational prodrug design has become an integrated and increasingly innovative part of drug discovery efforts, facilitating improved targeting of therapeutics through the generation of inactive analogues which release the active drug upon specific activation at the tumour site.^2^ In particular, the incorporation of masking groups which block the reactivity of important functional groups and are cleavable upon activation by enzymes overexpressed in the tumour microenvironment are increasingly being used in prodrug strategies, offering rapid and specific release upon reaction at the tumour site.^3^ Glycosidase-activated prodrugs such as those which exploit the activity of β-galactosidase have previously been developed,^4^ typically consisting of a small molecule anticancer drug conjugated to a β-galactose moiety *via* a self-immolative linker (Fig. 1A).^5^ Enzymatic activation hydrolyses the glycosidic bond, triggering release of the active drug.

**Fig. 1:**
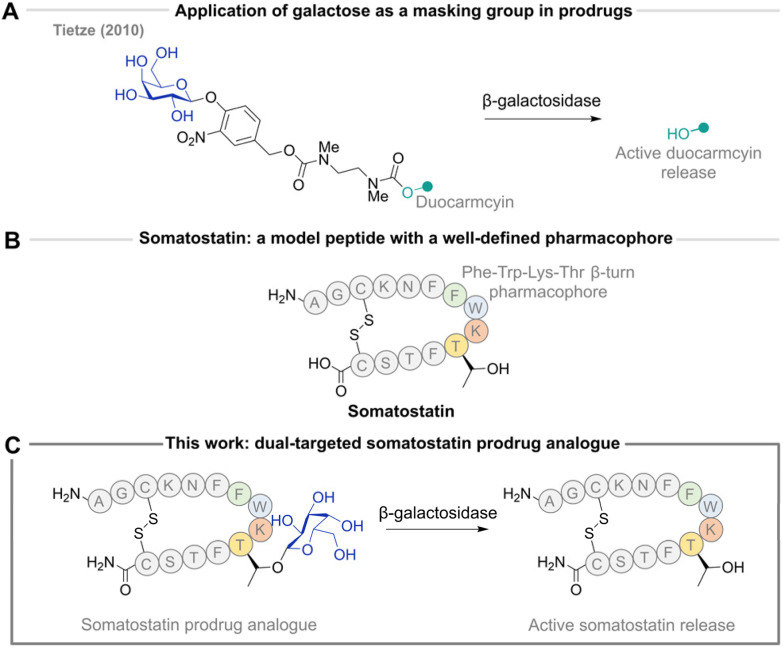
(A) Application of β-galactose masking groups to generate small molecule prodrugs; (B) the pharmacophore of somatostatin, highlighted in colour; (C) this work: application of a β-galactose masking group to the pharmacophore of somatostatin to generate a prodrug analogue, selectively activated by β-galactosidase.

Cyclic peptides have received growing attention as candidates for drug development due to their enhanced binding affinity, selectivity, and membrane permeability compared to their linear counterparts.^6,7^ Cyclic peptide hormone somatostatin together with its synthetic analogues bind specifically to five subtypes of somatostatin receptors (SSTRs) which are upregulated in many cancers, making them suitable targeting ligands for tumour-targeted peptides in addition to their use in the clinic as therapeutics.^8^ The application of these analogues to peptide–drug conjugates with small molecule anticancer agents and radiolabelled chelating agents has facilitated targeted delivery to cancer cells by exploiting the binding of the peptide to these receptors.^9,10^ In recent years, enzymatic activation of cleavable linkers for self-immolative drug release using tumour-homing peptides has been explored, offering a dual-targeting approach by exploiting the overexpression of both the peptide receptors and the enzyme.^11,12^ Despite advances in the development of bioactive peptides, systemic release and off-target effects can remain a limitation to their efficacy,^13^ as well as unsatisfactory metabolic stability.^14,15^ Systemic release of somatostatin analogues specifically has been associated with off-target cardiac effects^16^ and gallbladder abnormalities.^17^ Addressing these challenges through the development of therapeutic peptides as inactive prodrugs which are released in a targeted manner has remained an underexplored field.

Somatostatin has a well-defined pharmacophore which is constituted by the Phe^7^-Trp^8^-Lys^9^-Thr^10^ β-turn (Fig. 1B).^18^ β-galactosidase is overexpressed in a range of tumour types including gastric cancers which also exhibit upregulation of somatostatin receptors.^19,20^ In this work, we invisaged the application of a β-galactose masking group located on the threonine amino acid in the pharmacophore would generate a more stable, inactive analogue of the therapeutic peptide, suitable for use as a prodrug. Here, we present a glycopeptide somatostatin analogue, with the threonine residue of the pharmacophore masked with a β-galactose moiety, as a model system for the generation of activatable prodrug peptide derivatives (Fig. 1C). This strategy involves the extracellular β-galactosidase-mediated release of the active peptide which is subsequently able to bind to its cell-surface G protein-coupled receptors in a targeted manner.

To initiate our study, we developed a synthetic route to glycoamino acid 1 (Scheme 1). Firstly, an allyl ester threonine derivative suitable for use as an acceptor in a glycosylation reaction was required. Fmoc-Thr(^*t*^Bu)-OH 2 was allylated with allyl bromide to provide Fmoc-Thr(^*t*^Bu)-OAllyl 3 in 94% yield, followed by quantitative TFA-mediated removal of the *tert*-butyl side-chain protecting group to furnish acceptor Fmoc-Thr-OAllyl 4. Next, hemiacetal 6 was generated through reaction of peracetylated galactose 5 with benzylamine in 76% yield, which was subsequently reacted with trichloroacetonitrile and Cs_2_CO_3_ to give trichloroacetimidate 7 as a separable anomeric mixture (9 : 1 *α* : *β*) in a combined quantitative yield. Schmidt glycosylation with Fmoc-Thr-OAllyl 4 using BF_3_·Et_2_O as a Lewis acid catalyst furnished β-galactoside 8 in a 58% yield. Finally, deprotection of the allyl group *via* Pd-mediated deallylation provided glycoamino acid 1 in a yield of 92%. The ^3^*J*_H1,H2_ coupling constant of 8.0 Hz observed in the ^1^H NMR spectrum of 1, characteristic of a 180° axial–axial proton coupling in accordance with the Karplus equation,^21^ confirmed a diastereomerically pure β-galactoside. Overall, glycoamino acid 1 was prepared in an excellent yield of 38% over 6 steps.

**Scheme 1:**
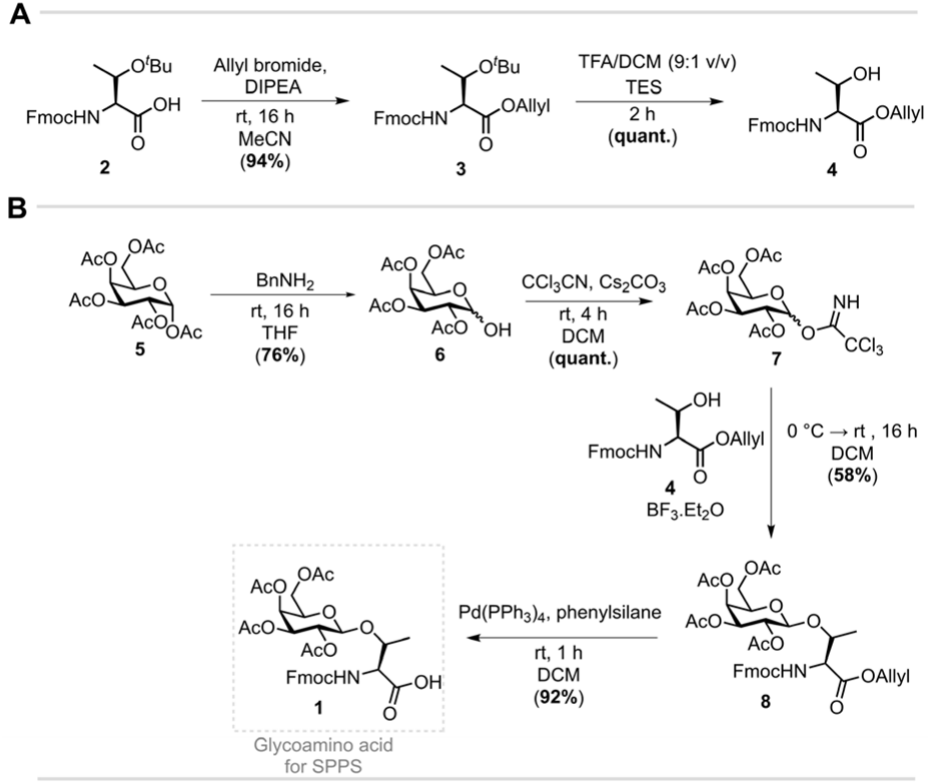
(A) Synthesis of threonine acceptor Fmoc-Thr-OAllyl 4. (B) Synthesis of glycoamino acid threonine analogue 1.

Typically, glycosidase-activated prodrugs incorporate a self-immolative linker within the structure which, upon glycosidase-mediated hydrolysis of the glycosidic bond, breaks down *via* a reaction cascade process to release the active drug.^22,23^ However, the disassembly of these linkers can generate side products such as quinone methide derivatives which have been associated with off-target cytotoxicity through DNA alkylation.^24^ Hence, glycoamino acid 1 was designed such that no linker would be required, eliminating unwanted side-product generation upon prodrug activation and reducing synthetic complexity.

Having generated glycoamino acid 1, we next synthesised a glycopeptide analogue of somatostatin, 9, *via* Fmoc solid-phase peptide synthesis (SPPS) using Rink Amide resin (Scheme 2). HATU/DIPEA in DMF was used for all amino acid couplings, except for the coupling of cysteine, which was coupled using DIC/Oxyma Pure in DMF : DCM (1 : 1 v/v). On-resin deacetylation was performed in order to minimise the number of peptide manipulation and purification steps required following peptide cleavage. Deacetylation was achieved using a solution of hydrazine hydrate (51% aq.) in DMF (1 : 1 v/v), previously reported by Galashov *et al.*,^25^ furnishing the deacetylated glycopeptide prior to cleavage from the resin. Peptide cleavage using TFA : EDT : H_2_O : TES (94 : 2.5 : 2.5 : 1 v/v) provided linear glycopeptide S1 in 30% yield. The linear peptide was cyclised at 1 mM concentration over 48 h at rt using DMSO as the oxidising agent, followed by purification by semi-preparative HPLC and lyophilisation to provide glycopeptide somatostatin analogue 9 in an isolated yield of 11%. The corresponding somatostatin analogue with a canonical threonine residue was synthesised using the same approach, with the linear precursor S2 isolated in 61% yield and the cyclic peptide 10 in 9% yield following oxidation and purification (Fig. 2).

**Scheme 2:**
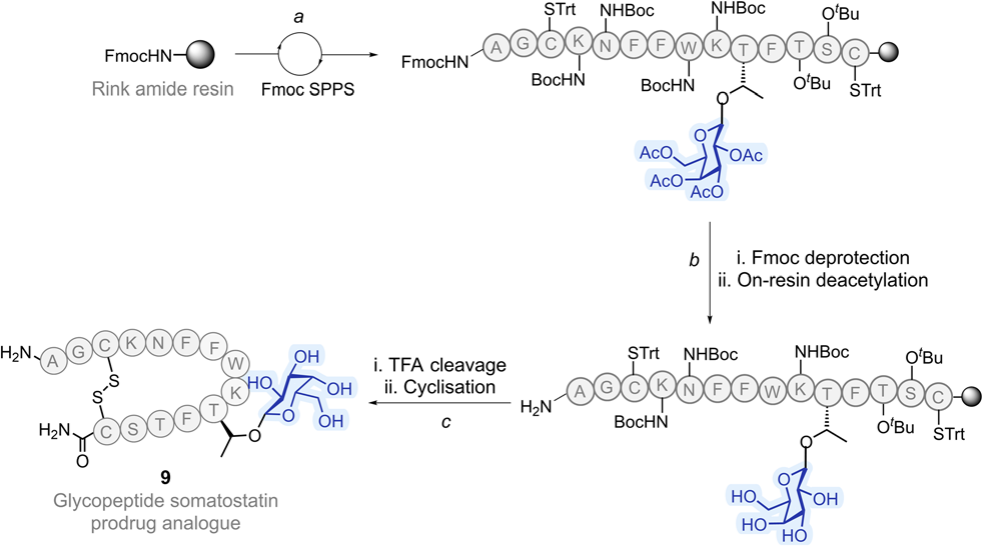
Solid phase peptide synthesis of glycopeptide 9. (a) Resin deprotection: 20% (v/v) piperidine in DMF, 2 × 10 min; standard coupling: Fmoc-AA-OH (4.0 eq.), HATU (3.9 eq.), DIPEA (8.0 eq.) in DMF, 45 min, cysteine coupling: Fmoc-Cys(Trt)-OH (4.0 eq.), DIC (4.0 eq.), Oxyma Pure (4.0 eq.), 45 min; deprotection: 20% (v/v) piperidine in DMF, 2 × 10 min (b) Fmoc deprotection: 20% (v/v) piperidine in DMF, 2 × 10 min; deacetylation: hydrazine hydrate (51% aq.) in DMF (1 : 1 v/v), 30 min. (c) Cleavage: TFA : EDT:H_2_O : TES (94 : 2.5 : 2.5 : 1 v/v); cyclisation: 10% (v/v) DMSO, 48 h.

**Fig. 2:**
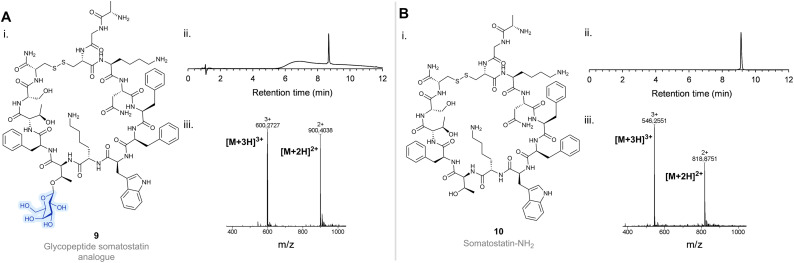
(A) (i) Structure of glycopeptide somatostatin analogue 9 (ii) HPLC trace (220 nm), *R*_T_ = 8.68 min (iii) ESI mass spectrum corresponding to this peak. (iii) ESI mass spectrum of this peak (B) (i) structure of somatostatin-NH_2_10 (ii) HPLC trace (220 nm), *R*_T_ = 9.15 min.

With our glycopeptide somatostatin analogue 9 and somatostatin-NH_2_10 in hand, we next sought to evaluate their cytotoxicity against cancer cells in order to validate the suitability of glycopeptide 9 as an activatable prodrug. An MTT (3-(4,5-dimethylthiazol-2-yl)-2,5-diphenyltetrazolium bromide) assay using HeLa cervix carcinoma cells were employed for this cytotoxicity study due to their high expression levels of SSTR2.^26^ SSTR2 is the receptor subtype overexpressed in the largest number of tumour types and to which somatostatin and its analogues demonstrate their highest affinity.^27^ In addition, β-galactosidase is not naturally upregulated in this cell line under standard culture conditions; it is a known marker of senescence, only becoming overexpressed at pH 6.0 in senescent cells.^28,29^ The low β-galactosidase expression levels allowed us to design the assay such that the addition of β-galactosidase could validate an increase in cytotoxicity upon extracellular β-galactosidase-mediated release of the active peptide following removal of the β-galactose masking group. The combination of high SSTR2 expression and low β-galactosidase expression in HeLa cells therefore affords the ideal conditions to test glycopeptide 9 and its release to somatostatin-NH_2_10. Isopropyl β-D-1-thiogalactopyranoside (IPTG) was used as a competitive β-galactosidase inhibitor to validate any observed enzyme response.

The MTT assay was performed after 72 h, measuring HeLa cell metabolic activity following treatment with only glycopeptide 9 (33 µM), with the addition of β-galactosidase (100 U mL^−1^) and finally with the addition of both β-galactosidase and inhibitor IPTG (Fig. 3, glycopeptide 9). The same assay was carried out using somatostatin-NH_2_10 (Fig. 3, somatostatin-NH_2_10). Gratifyingly, the results showed higher metabolic activity of the cells treated with glycopeptide 9 relative to the somatostatin-NH_2_10, indicated by a rise in optical density from 1.06 to 1.23 of 16%. Moreover, the assay showed a significant β-galactosidase-mediated decresase in metabolic activity for the cells treated with glycopeptide 9 upon enzyme treatment, indicated by a reduction in optical density from 1.23 to 0.80 of 35%. When cells were treated with β-galactosidase inhibitor IPTG in addition to glycopeptide 9 and β-galactosidase, a smaller decrease in metabolic activity compared to the untreated peptide was observed as the optical density reduced from 1.23 to 0.99, indicating a reduced release of the active peptide following enzyme inhibition. In addition, for HeLa cells that had been treated with glyocpeptide 9, the optical density following treatment with IPTG only was also found to be 0.99 (Fig. S1), confirming inhibition of the enzymatic response. Whilst control experiments in cell media with no peptide exhibited a small reduction in metabolic activity on enzyme treatment, this effect was limited and IPTG did not demonstrate inhibition of this effect (Fig. S1).

**Fig. 3:**
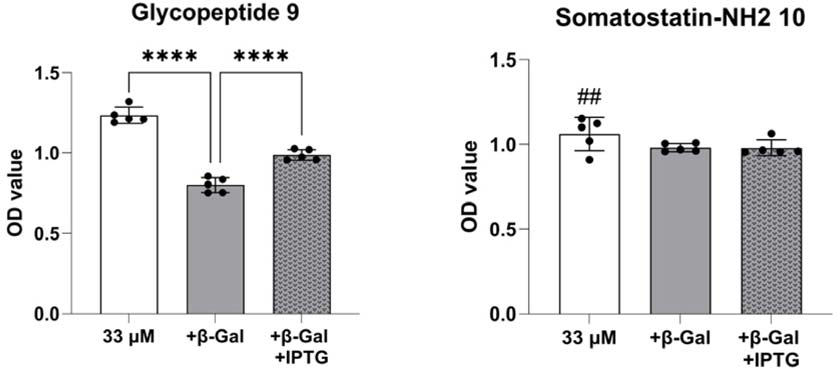
Comparison of glycopeptide 9 and somatostatin-NH_2_10 responses in HeLa metabolic activity by MTT assay. HeLa metabolic activity was determined by MTT assay (*n* = 5) following 72 h treatment with glycopeptide 9 or somatostatin-NH_2_10 (33 µM) under different conditions: (i) glycopeptide 9 or somatostatin-NH_2_10 (33 µM) alone, (ii) with 100 U mL^−1^ β-galactosidase (+β-Gal), and (iii) with 100 U mL^−1^ β-galactosidase and 0.2 mM β-galactosidase inhibitor IPTG (+ β-Gal + IPTG). Results are presented as mean optical density (O.D.) ± standard deviation. Statistical analysis was performed using one-way ANOVA. Significance between different conditions in the same group is indicated as follows: *p* < 0.0001 (****). Significance compared to control is indicated as follows: *p* < 0.01 (##).

For cells treated with the somatostatin-NH_2_10, without a β-galactosidase-cleavable masking group on the pharmacophore of the peptide a statistically insignificant difference in metabolic activity was observed on β-galactosidase treatment. Likewise, treatment with IPTG in addition to somatostatin-NH_2_10 and β-galactosidase resulted in a negligible effect on the cells. Treatment of peptide 10 with IPTG only also demonstrated a small cytotoxic effect (Fig. S1). For glycopeptide 9 to act as a conventional activatable prodrug, it would be expected for enzymatic activation to reduce the level of metabolic activity to that of the cells treated with active compound. However, the optical density of cells treated with glycopeptide 9 following β-galactosidase treatment of 0.80 corresponds to a 25% decrease in metabolic activity compared to untreated somatostatin-NH_2_10 which had an optical density of 1.06, indicating improved potency of the glycopeptide. A widely reported limitation of somatostatin that has precluded its use in the clinic is its prohibitively short half-life of 1 to 3 minutes and instability.^15,30^ Glycoconjugation has previously been reported as a strategy for increasing the metabolic stability and resistance to protease degradation of therapeutic peptides^31–33^ Glycosylated analogues of peptide hormones including somatostatin have demonstrated improved metabolic stability compared to the native peptide, protecting the peptide from enzymatic digestion.^34,35^ Moreover, glycoamino acid 1 replaces the threonine residue of a key endopeptidase cleavage site at Thr^10^-Phe^11^.^36^

In addition, one of the primary purposes of prodrug development utilising masking groups is to impart increased chemical and enzymatic stability to the parent drug.^2^ This strategic approach is designed to reduce degradation and increase relative accumulation of the active form at the target site, leading to a prolonged treatment time in comaprison to the native peptide thus enhancing the cytotoxicity of the administered drug at this target. As such, we postulate that increased stability of glycopeptide 9 is imparted through the masking of the pharmacophore, resulting in a prolonged duration of action at its target SSTRs, manifesting an increased cytotoxic effect to a level that exceeds that of the native peptide. These results indicate the suitability of glycopeptide 9 as a masked prodrug analogue of therapeutic peptide somatostatin. The specific activation of glycopeptide 9 by β-galactosidase, which is upregulated in the microenvironment of various tumours, causes a marked increase in cytotoxicity in a β-galactosidase-selective fashion.

## Conclusions

In summary, we have described the synthesis of an unnatural glycoamino acid 1 and its incorporation *via* SPPS to generate a glycopeptide somatostatin analogue 9, designed as a model peptide prodrug for selective activation by β-galactosidase. The glycopeptide was found to exhibit a reduced cytotoxicity compared to somatostatin and demonstrated a significant β-galactosidase-mediated increase in cytotoxicity to a level that exceeded that of somatostatin. We hypothesise that these observations are as a result of the binding of the released somatostatin to the SSTR2 receptor, although this has not been confirmed by a direct binding assay. This represents a new application of an activatable prodrug strategy to a therapeutic peptide as a way to improve the targeted release and potency of the peptide. The extension of this strategy to other bioactive peptides through masked pharmacophores and to more complex systems such as peptide–drug conjugates which utilise tumour-homing peptides opens the potential for a new class of therapeutics with improved overall efficacies.

## Author contributions

Conceptualisation: J. F. M.; methodology: C. J. O., J. F. M.; investigation: C. J. O., S. C. , A. N. , A. B.; data curation and visualisation: C. J. O., S. C.; writing – original draft: C. J. O.; writing – review and editing: C. J. O., J. F. M., M. G. M., E. M. S, S. C; supervision: J. F. M., M. G. M.; funding acquisition: J. F. M.; resources: J. F. M., M. G. M., E. M. S., K. K.

## Conflicts of interest

There are no conflicts to declare.

## Supplementary Material

RA-OLF-D5RA08677A-s001

## Data Availability

The data supporting this article have been included as part of the supplementary information (SI). Supplementary information: full characterisation data for all compounds (including NMR spectra, RP-HPLC traces), synthetic chemistry experimental procedures, biological assay experimental procedures and figures. See DOI: https://doi.org/10.1039/d5ra08677a.
