# Supplementary material for: Design, synthesis and biological evaluation of a β-galactosidase-activated glycopeptide analogue of somatostatin
Source: RSC Adv. 2026 Jul 21. Online ahead of print. doi: 10.1039/d5ra08677a (PMC13386423; doi:10.1039/d5ra08677a)
Supplement: RA-OLF-D5RA08677A-s001 [file RA-OLF-D5RA08677A-s001.pdf]

Supplementary Information (SI) for RSC Advances.
This journal is © The Royal Society of Chemistry 2026

Supporting Information

Design, synthesis and biological evaluation of a β-galactosidase-activated glycopeptide analogue of somatostatin

Connor J. O'Leary,[ab] Sara Corbezzolo,[c] Ailish Newton,[a] Alby Benny,[a] Eoin M. Scanlan,[a] Krystian Kolodziejczak,[d] Michael G. Monaghan[c] and Joanna F. McGouran[*ab]

[a]Trinity Biomedical Sciences Institute, School of Chemistry, Trinity College Dublin, 152-160 Pearse St, D02 R590, Ireland.

[b]SSPC, the Research Ireland centre for pharmaceuticals.

[c]Department of Mechanical, Manufacturing & Biomedical Engineering, Trinity College Dublin, Dublin 2.

[d]Ringaskiddy Active Pharmaceutical Ingredient Plant, Pfizer Ireland Pharmaceuticals, Ringaskiddy, Co. Cork, Ireland

*Please direct correspondence to jmcgoura@tcd.ie.

Contents

**S2**	General Experimental Methods
**S4**	Synthesis of Glycoamino Acid **1**
**S11**	General Procedures for Solid-Phase Peptide Synthesis (SPPS)
**S13**	Synthesis of Peptides
**S17**	Biological Methods
**S19**	NMR Spectra
**S33**	References

General Methods

Chemical Synthesis

Reagents used were purchased from Sigma Aldrich, Fisher Scientific, Fluorochem and Tokyo Chemical Industry (TCI) and used without further purification. All moisture-sensitive reactions were carried out in oven-dried round bottom-flasks under an argon atmosphere in anhydrous solvents. Anhydrous DCM, THF and MeCN were dried using a PureSolv MD solvent purification system and anhydrous DMF, MeOH and EtOH were purchased from Sigma Aldrich and dried over molecular sieves. Petroleum ether refers to the fraction of petroleum ether that boils at 40 – 60 °C. Room temperature (rt) reactions were carried out between 18 – 21 °C. Melting points were measured using a Griffin melting point apparatus and are uncorrected with solvents of crystallisation listed along with the observed melting point range.

Flash Column Chromatography and Thin Layer Chromatography (TLC)

Flash column chromatography was performed with 60 Å (230-400 mesh particle size) silica gel from Sigma Aldrich. TLC was conducted using aluminium backed plates coated with silica matrix (0.2 mm) and fluorescent indicator (254 nm) from Sigma Aldrich. Visualisation was carried out using a UV lamp and/or appropriate staining with ninhydrin (1.5 g ninhydrin, 5 mL AcOH, 500 mL 95% EtOH) and anisaldehyde (9.2 mL *p*-methoxybenzaldehyde, 3.75 mL AcOH, 338 mL 95% EtOH).

High-Resolution Mass Spectrometry (HRMS)

High resolution mass spectra were acquired by ESI and APCI using a Bruker microTOF-Q III mass spectrometer (set to EI^+ or EI^- mode where appropriate). ESI mass spectra were acquired using a Bruker micrOTOF-Q III spectrometer interfaced to a Dionex UltiMate 3000 LC in positive and negative modes as required. Agilent ESI-L Low Concentration Tuning Mix was used to calibrate the system, and this was also used as an internal lock mass. Masses were recorded over the range 100 – 2000 m/z. Operating conditions were as follows: end-plate offset 500 V, capillary 4500 V, nebulizer 2.0 bar, dry gas 8.0 L min^{-1}, and dry temperature 180 °C.

APCI experiments were carried out on a Bruker micrOTOF-Q III spectrometer interfaced to a Dionex UltiMate 3000 LC or direct insertion probe in positive or negative mode as required. Agilent APCI-TOF Tuning Mix was used to calibrate the system. Masses were recorded over a range of 100 – 2000 *m/z*. Operating conditions were as follows: capillary voltage 4000 V, corona 4000 nA, nebulizer gas 2.0 bar, dry gas 3.0 L min^{-1}, dry gas temperature 100 – 200 °C, vap. temperature 100

– 400 °C. Compass HyStar version 3.2 and micrOTOF Control version 3.2 software were used to analyse the data.

Nuclear Magnetic Resonance (NMR) Spectroscopy

NMR spectra were recorded on either an Agilent MR 400 spectrometer with operating frequencies of 399.84 MHz for ^{1}H and 100.55 MHz for ^{13}C, a Bruker Avance III 400 spectrometer with operating frequencies of 400.23 MHz for ^{1}H and 100.63 MHz for ^{13}C, or a Bruker Avance II 600 spectrometer with operating frequencies of 600.13 MHz for ^{1}H and 150.90 MHz for ^{13}C. NMR spectra were processed using MestReNova (version 14.2.2). ^{1}H NMR chemical shifts were referenced to residual non-deuterated solvent peaks within the NMR solvent; $CHCl_3$ (δ_H = 7.26 ppm) and DMSO (δ_H = 2.50 ppm). The chemical shifts (δ) are reported in parts per million (ppm) and signal multiplicity as s = singlet, d = doublet, t = triplet, q = quartet, m = multiplet and combinations thereof. Coupling constant (*J*) are quoted in Hertz (Hz) and reported to the nearest 0.1 Hz. ^{13}C NMR spectra are proton-decoupled with chemical shifts referenced to residual deuterated solvent peaks in the NMR solvent; $CDCl_3$ (δ_C = 77.16 ppm) and DMSO-d_6 (δ_C = 39.52 ppm). Product identification and peak assignments were carried out using additional 1D experiments (TOCSY), 2D experiments (HSQC, HMBC) and pulse experiments (DEPT-135) where appropriate.

Infra-red (IR) Spectroscopy

Infra-red spectra were acquired using a Perkin Elmer FT-IR Spectrum 100 spectrometer equipped with an ATR attachment. Absorbance shape and intensity are described as w = weak; m = medium; s = strong; sh = sharp; br = broad.

High-Performance Liquid Chromatography (HPLC)

Reverse phase (RP) high performance liquid chromatography (HPLC) was performed on a Shimadzu Nexera lite Low Pressure Gradient system with an SPD-M40 photodiode array (PDA) detector at 220 nm. For analytical HPLC, an Ascentis Express 90 Å C18, 100 mm x 4.6 mm; 5 µm column with a flow rate of 1 mL min^{-1}. A gradient of 5% solvent B to 95% was used (solvent A: 0.1% TFA in H_2O; solvent B: 0.1% TFA in MeCN).

For semi-preparative HPLC purification, an Ascentis C18 250 x 10 mm, 5 µm column at a flow rate of 4 mL min^{-1} and eluted using a gradient of 5% solvent B to 95% over 30 min followed by 95% solvent B for 10 min. Fractions were collected, lyophilised, and analysed by RP-HPLC and HRMS.

Synthesis of Glycoamino Acid 1

Fmoc-Thr(^{t}Bu)-OAllyl (3)

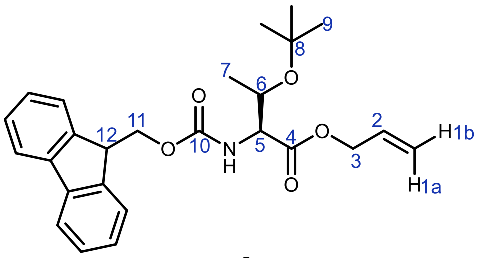

3

Allyl bromide (0.98 mL, 11.3 mmol) was added dropwise to a solution of Fmoc-Thr(^{t}Bu)-OH (3.00 g, 7.55 mmol) and DIPEA (1.97 mL, 11.3 mmol) in anhydrous MeCN (10 mL) and stirred at rt for 16 h under argon. After this time, TLC analysis (10% EtOAc/hexane) showed consumption of the starting material (R_F = 0.0) and the formation of a product (R_F = 0.3). The reaction mixture was then concentrated under reduced pressure and filtered through a silica plug (10% EtOAc/hexane) to give **3** (3.09 g, 7.07 mmol, 94%) as a clear oil.

^{1}H NMR (400 MHz, $CDCl_3$) δ_H 7.79 (app. dq, *J* 7.6 1.0 Hz, 2H, ArH), 7.66 (app. tq, *J* 7.6 1.0 Hz, 2H, ArH), 7.43 (tt, *J* 7.5 1.3 Hz, 2H, ArH), 7.35 (tt, *J* 7.5 1.3 Hz, 2H, ArH), 5.95 (ddt, *J* 17.2 10.4 5.8 Hz, 1H, H2), 5.64 (d, *J* 9.6 Hz, 1H, NH), 5.38 (app. dq, *J* 17.2 1.5 Hz, 1H, H1a), 5.29 (app. dp, *J* 10.3 1.3 Hz, 1H, H1b), 4.71 (ddt, *J* 13.1 5.8 1.5 Hz, 1H, H3a), 4.58 (ddt, *J* 13.1 5.8 1.5 Hz, 1H, H3b), 4.35 – 4.48 (m, 2H, H11), 4.24 – 4.33 (m, 3H, H5, H6, H12), 1.27 (d, *J* 6.2 Hz, 3H, H7), 1.17 (s, 9H, H9) ppm.

^{13}C NMR (101 MHz, $CDCl_3$) δ_C 170.9 (C4), 156.8 (C10), 144.1 (Fmoc qC), 143.9 (Fmoc q<u>C</u>), 141.3 (2x Fmoc q<u>C</u>), 131.6 (C2), 127.7 (Fmoc ArC), 127.10 (Fmoc ArC), 127.08 (Fmoc ArC), 125.3 (Fmoc ArC), 125.2 (Fmoc ArC), 120.0 (Fmoc ArC), 119.9 (C1), 74.1 (C8), 67.4 (C6), 67.3 (C11), 66.0 (C3), 60.0 (C5), 47.2 (C12), 28.4 (C9), 21.0 (C7) ppm.

HRMS (ESI^+) *m/z* calc. for $C_{26}H_{31}NNaO_5$ $[M+Na]^+$ 460.2094; found 460.2097.

The spectral data are in agreement with the literature.[1]

Fmoc-Thr-O(Allyl) (4)

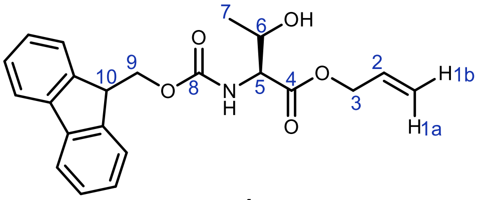

4

A solution of **3** (3.10 g, 7.09 mmol) in TFA:TES:DCM(2:1:8, v/v, 20 mL) at rt for 2 h. After this time, TLC analysis (10% EtOAc/hexane) showed consumption of the starting material (R_F = 0.3) and the formation of a new product spot (R_F = 0.0); analysis in 40% EtOAc/hexane confirmed consumption of the starting material (R_F = 0.9) and the formation of a product (R_F = 0.3). The solvent was removed under reduced pressure, coevaporated with MeOH (3 x 20 mL) and filtered through a silica plug (40% EtOAc/hexane) to give **4** (2.70 g, 7.08 mmol, quant.) as a clear oil.

^{1}H NMR (400 MHz, DMSO-d$_6$) δ_H 7.90 (d, *J* 7.5 Hz, 2H, ArH), 7.75 (ddd, *J* 7.5 2.1 1.0 Hz, 2H, ArH), 7.42 (t, *J* 7.5 Hz, 2H, ArH), 7.39 – 7.45 (m, 2H, ArH), 5.90 (ddt, *J* 17.4 10.5 5.2 Hz, 1H, H2), 5.34 (app. dq, *J* 17.4 1.7 Hz, 1H, H1a), 5.20 (app. dq, *J* 10.5 1.5 Hz, 1H, H1b), 4.83 (d, *J* 6.9 Hz, 1H, OH), 4.58 – 4.61 (m, 2H, H3), 4.29 – 4.33 (m, 2H, H9), 4.24 (t, *J* 7.0 Hz, 1H, H10), 4.07 – 4.14 (m, 2H, H5, H6), 1.11 (d, *J* 6.0 Hz, H7) ppm.

^{13}C NMR (101 MHz, DMSO-d$_6$) δ_C 170.5 (C4), 156.4 (C8), 143.8 (Fmoc qC), 143.7 (Fmoc qC), 140.7 (Fmoc qC), 132.4 (<u>C2</u>), 127.6 (Fmoc ArC), 127.0 (Fmoc ArC), 125.3 (Fmoc ArC), 120.1 (Fmoc ArC), 117.5 (C1), 66.4 (C6), 65.8 (C9), 64.8 (O<u>C</u>H$_2$CH), 60.2 (C5), 46.6 (C10), 20.1 (C7) ppm.

HRMS (ESI$^+$) *m/z* calc. for $C_{22}H_{24}NO_5$ $[M+H]^+$ 382.1649; found 382.1649.

The spectral data are in agreement with the literature.[1]

2,3,4,6-tetra-*O*-acetyl-α/β-D-galactopyranose (6)

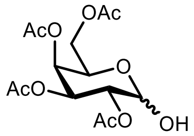

6

Benzylamine (4.19 mL, 38.5 mmol) was added to a solution of peracetylated galactose **5** (10.0 g, 25.63 mmol) in anhydrous THF (30 mL) and the reaction mixture was stirred for 16 h at rt under argon. After this time, TLC analysis (50% EtOAc/hexane) showed consumption of the starting material (R_F = 0.6) and the formation of a product (R_F = 0.3). The reaction mixture was concentrated under reduced pressure and the crude material purified by flash column chromatography (30 → 50% EtOAc/hexane) to give **6** (6.73 g, 19.33 mmol, 76%, 2:1 ratio α:β anomers) as a brown oil.

^{1}H NMR (400 MHz, CDCl$_3$) δ_H 5.53 (d, $J_{\alpha\text{-H1H2}}$ 3.6 Hz, 1H, α-H1), 5.48 (dd, $J_{\alpha\text{-H4H3}}$ 3.4 $J_{\alpha\text{-H4H5}}$ 1.4 Hz, 1H, α-H4), 5.42 – 5.44 (m, 1H, β-H3), 5.39 – 5.41 (m, 1H, α-H3), 5.17 (dd, $J_{\alpha\text{-H2H3}}$ 10.8 Hz $J_{\alpha\text{-H2H1}}$ 3.6 Hz, 1H, α-H2), 5.06 – 5.09 (m, 2H, β-H2, β-H4), 4.70 – 4.73 (m, 1H, β-H1), 4.47 (td, $J_{\alpha\text{-H5H6}}$ 6.6 Hz $J_{\alpha\text{-H5H4}}$ 1.4 Hz, 1H, α-H5), 4.05 – 4.16 (m, 4H, α-H6, β-H6), 3.95 (td, $J_{\beta\text{-H5H6}}$ 6.6 $J_{\beta\text{-H5H4}}$ 1.2 Hz, 1H, β-H5), 2.16 (s, 3H, β-OCOC$\underline{H}_3$), 2.15 (s, 3H, α-OCOC$\underline{H}_3$), 2.11 (s, 3H, β-OCOC$\underline{H}_3$), 2.10 (s, 3H, α-OCOC$\underline{H}_3$), 2.07 (s, 3H, β-OCOC$\underline{H}_3$), 2.05 (s, 3H, α-OCOC$\underline{H}_3$), 2.01 (s, 3H, β-OCOC$\underline{H}_3$), 2.00 (s, 3H, α-OCOC$\underline{H}_3$) ppm.

^{13}C NMR (101 MHz, CDCl$_3$) δ_C 171.4 (O$\underline{C}$OCH$_3$),170.52 (O$\underline{C}$OCH$_3$), 170.48 (O$\underline{C}$OCH$_3$), 170.4 (O$\underline{C}$OCH$_3$), 170.24 (O$\underline{C}$OCH$_3$),170.16 (O$\underline{C}$OCH$_3$), 170.1 (O$\underline{C}$OCH$_3$), 170.0 (O$\underline{C}$OCH$_3$), 96.1 (β-C1), 90.7 (α-C1), 71.13 (β-C5), 71.06 (β-C4), 70.3 (β-C2), 68.3 (α-C2), 68.2 (α-C4), 67.2 (β-C3), 67.1 (α-C3), 66.3 (α-C5), 61.8 (α-C6), 61.5 (β-C6), 20.8 (OCO$\underline{C}$H$_3$), 20.74 (OCO$\underline{C}$H$_3$), 20.72 (OCO$\underline{C}$H$_3$), 20.67 (OCO$\underline{C}$H$_3$), 20.65 (OCO$\underline{C}$H$_3$), 20.63 (OCO$\underline{C}$H$_3$), 20.57 (OCO$\underline{C}$H$_3$), 20.4 (OCO$\underline{C}$H$_3$) ppm.

HRMS (ESI$^+$) *m/z* calc. for $C_{14}H_{20}NaO_{10}$ [M+Na]$^+$ 371.0949; found 371.0951.

The spectral data are in agreement with the literature.[2]

1-*O*-(2,3,4,6-tetra-O-acetyl-α-D-galactopyranosyl)trichloroacetimidate (7α)

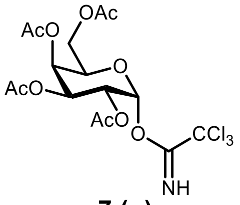

7 (α)

CCl_3CN (11.52 mL, 114.9 mmol) and Cs_2CO_3 (936 mg, 2.87 mmol) were added to a solution of **6** (4.00 g, 11.49 mmol) in anhydrous DCM (30 mL) and stirred for 4 h at rt under argon. After this time, TLC analysis (30% EtOAc/hexane) showed partial consumption of the starting material (R_F = 0.1) and the formation of two new products (R_F = 0.5). The reaction mixture was concentrated under reduced pressure and the crude product purified by flash column chromatography (20% → 30% EtOAc/hexane) to give **7α** (5.03 g, 10.25 mmol, 89%) as an off-white crystalline solid, along with **7β** (621 mg, 1.26 mmol, 11%) as an off-white foam.

^{1}H NMR (400 MHz, $CDCl_3$) δ_H 8.66 (s, 1H, NH), 6.60 (d, J_{H1H2} 3.5 Hz, 1H, H1), 5.56 (dd, J_{H4H3} 3.1 Hz J_{H4H5} 1.3 Hz, 1H, H4), 5.43 (dd, J_{H3H2} 10.9 J_{H3H4} 3.1 Hz, 1H, H3), 5.36 (dd, J_{H3H2} 10.9 J_{H2H1} 3.5 Hz, 1H, H2), 4.44 (dt, J_{H5H6} 6.6 J_{H5H4} 1.3 Hz, 1H, H5), 4.17 (dd, J_{H6aH6b} 11.3 J_{H6aH5} 6.6 Hz, 1H, H6a), 4.08 (dd, J_{H6bH6a} 11.3 J_{H6bH5} 6.6 Hz, 1H, H6b), 2.17 (s, 3H, OCOC$\underline{H}_3$), 2.03 (s, 3H, OCOC$\underline{H}_3$), 2.02 (s, 3H, OCOC$\underline{H}_3$), 2.01 (s, 3H, OCOC$\underline{H}_3$) ppm.

^{13}C NMR (101 MHz, $CDCl_3$) δ_C 170.3 (O$\underline{C}$OCH$_3$),170.12 (O$\underline{C}$OCH$_3$), 170.09 (O$\underline{C}$OCH$_3$), 170.0 (O$\underline{C}$OCH$_3$), 161.0 (O$\underline{C}$NH), 93.6 (C1), 90.8 ($\underline{C}$Cl$_3$), 69.0 (C5), 67.5 (C4), 67.4 (C3), 66.9 (C2), 61.3 (C6), 20.7 (OCO$\underline{C}$H$_3$), 20.64 (OCO$\underline{C}$H$_3$), 20.62 (OCO$\underline{C}$H$_3$), 20.55 (OCO$\underline{C}$H$_3$) ppm.

HRMS (ESI$^+$) *m/z* calc. for $C_{16}H_{20}Cl_3KNO_{10}$ $[M+K]^+$ 529.9784; found 529.9788.

mp 121-122 °C (lit.[3] 122 – 123 °C) from EtOAc/hexane.

The spectral data are in agreement with the literature.[4]

1-*O*-(2,3,4,6-tetra-O-acetyl-α-D-galactopyranosyl)trichloroacetimidate (7β)

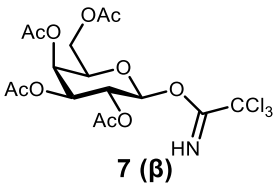

7 (β)

^{1}H NMR (400 MHz, $CDCl_3$) δ_H 8.71 (s, 1H, NH), 5.84 (d, J_{H1H2} 8.2 Hz, 1H, H1), 5.48 – 5.51 (m, 1H, H2), 5.44 – 5.47 (m, 1H, H4), 5.12 (dd, J_{H3H2} 10.4 J_{H3H4} 3.5 Hz, 1H, H3), 4.17 – 4.21 (m, 2H, H6), 4.08 – 4.13 (m, 1H, H5), 2.18 (s, 3H, OCOC$\underline{H}_3$), 2.04 (s, 3H, OCOC$\underline{H}_3$), 2.02 (s, 3H, OCOC$\underline{H}_3$), 2.01 (s, 3H, OCOC$\underline{H}_3$) ppm.

^{13}C NMR (101 MHz, $CDCl_3$) δ_C 170.3 (O$\underline{C}$OCH$_3$), 170.2 (O$\underline{C}$OCH$_3$), 170.1 (O$\underline{C}$OCH$_3$), 169.1 (O$\underline{C}$OCH$_3$), 161.1 (O$\underline{C}$NH), 96.1 (C1), 90.4 ($\underline{C}$Cl$_3$), 71.8 (C5), 70.7 (C3), 67.7 (C2), 66.7 (C4), 60.9 (C6), 20.7 (3x OCO$\underline{C}$H$_3$), 20.6 (OCO$\underline{C}$H$_3$) ppm.

HRMS (ESI$^+$) *m/z* calc. for $C_{16}H_{20}Cl_3NNaO_{10}$ $[M+Na]^+$ 514.0045; found 514.0048.

The spectral data are consistent with the literature.[5]

(allyl(((9H-fluoren-9-yl)methoxy)carbonyl)-L-threoninate

)-2,3,4,6-tetra-*O*-acetyl-β-D-galactopyranoside (8)

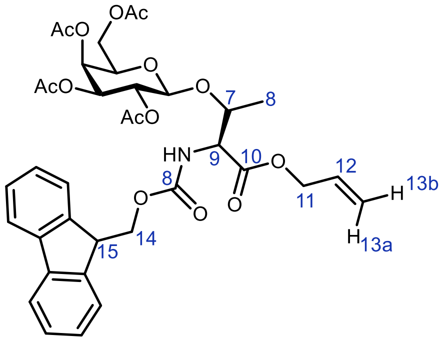

8

$BF_3 \cdot Et_2O$ (0.83 mL, 6.72 mmol) was added dropwise to a solution of trichloroacetimidate **7** (3.0 g, 6.11 mmol) and **4** (3.49 g, 9.17 mmol) in anhydrous DCM (20 mL) at 0 °C, allowed to warm to rt and stirred for 16 h under argon. After this time, TLC analysis (30% EtOAc/hexane) showed consumption of the starting material (R_F = 0.5) and formation of a product (R_F = 0.2), and the reaction was quenched with Et_3N (0.93 mL, 6.72 mmol) and concentrated under reduced pressure. The crude material was then purified by flash column chromatography (30% → 50% EtOAc/hexane) to give **8** (2.51 g, 3.53 mmol, 58%) as an off-white crystalline solid.

^{1}H NMR (400 MHz, $CDCl_3$) δ_H 7.78 (d, *J* 7.5 Hz, 2H, ArH), 7.67 (t, *J* 8.2 Hz, 2H, ArH), 7.42 (tt, *J* 7.6 1.3 Hz, 2H, ArH), 7.34 (td, *J* 7.5 1.3 Hz, 2H, ArH), 5.87 – 6.00 (m, 1H, H12), 5.63 (d, *J* 9.3 Hz, 1H,

NH), 5.33 – 5.42 (m, 2H, H4, H13a), 5.27 (app. dq, *J* 10.5 1.4 Hz, 1H, H13b), 5.15 (d, *J* 10.5 7.8 Hz, 1H, H2), 5.03 (dd, *J* 10.5 3.5 Hz, 1H, H3), 4.63 – 4.74 (m, 2H, H11), 4.35 – 4.51 (m, 5H, H14, H9, H7, H1), 4.28 (t, *J* 7.4 Hz, 1H, H15), 4.08 – 4.14 (m, 2H, H6), 3.88 (dt, *J* 6.8 1.3 Hz, 1H, H5), 2.17 (s, 3H, OCOC$\underline{H}_3$), 2.07 (s, 3H, OCOC$\underline{H}_3$), 2.06 (s, 3H, OCOC$\underline{H}_3$), 2.01 (s, 3H, OCOC$\underline{H}_3$), 1.21 – 1.24 (m, 3H, H8) ppm.

^{13}C NMR (101 MHz, $CDCl_3$) δ_C 170.4 (O$\underline{C}$OCH$_3$), 170.2 (O$\underline{C}$OCH$_3$), 170.1 (O$\underline{C}$OCH$_3$), 169.7 (O$\underline{C}$OCH$_3$), 169.4 (O$\underline{C}$OCH$_3$), 161.1 (C10), 156.8 (C8), 144.0 (Fmoc qC), 143.7 (Fmoc qC), 141.3 (Fmoc qC), 131.7 (Fmoc qC), 127.7 (Fmoc ArC), 127.12 (Fmoc ArC), 127.09 (Fmoc ArC), 125.3 (Fmoc ArC), 125.2 (Fmoc ArC), 120.0 (Fmoc ArC), 118.6 (C13), 99.5 (C1), 75.0 (C7), 70.7 (C3), 70.6 (C5), 68.8 (C2), 67.3 (C14), 66.8 (C4), 66.2 (C11), 60.9 (C6), 58.6 (C9), 47.2 (C15), 21.1 (OCO$\underline{C}$H$_3$), 20.8 (OCO$\underline{C}$H$_3$), 20.7 (OCO$\underline{C}$H$_3$), 20.6 (OCO$\underline{C}$H$_3$), 17.2 (C8) ppm.

HRMS (ESI$^+$) *m/z* calc. for $C_{36}H_{41}KNO_{14}$ $[M+K]^+$ 750.2159; found 750.2158.

ν_{max}/cm^{-1} (neat) 2981 (ArC), 1745 (CO).

mp 62-64 °C from EtOAc/hexane.

((((9H-fluoren-9-yl)methoxy)carbonyl)-L-threonine

)-2,3,4,6-tetra-*O*-acetyl-β-D-galactopyranoside (1)

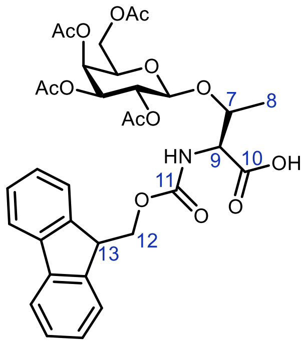

1

Phenylsilane (0.67 mL, 5.44 mmol) and $Pd(PPh_3)_4$ (16 mg, 0.0136 mmol) were added to a solution of **8** (968 mg, 1.36 mmol) in anhydrous DCM (10 mL) and stirred for 1 h at rt under argon. After this time, TLC analysis (40% EtOAc/hexane) showed complete consumption of the starting material (R_F = 0.3) and the formation of a product (R_F = 0.0); analysis in 5% MeOH/DCM confirmed consumption of the starting material (R_F = 1.0) and formation of a product (R_F = 0.3). The reaction mixture was concentrated under reduced pressure and the crude material purified by flash column chromatography (5% MeOH/DCM) to give **1** (841 mg, 1.25 mmol, 92%) as a pale brown crystalline solid.

^{1}H NMR (400 MHz, DMSO-d_6) δ_H 7.89 (d, *J* 7.5 Hz, 2H, ArH), 7.72 (dd, *J* 7.5 2.2 Hz, 2H, ArH), 7.42 (t, *J* 7.5 Hz, 2H, ArH), 7.33 (dd, *J* 8.3 6.4 Hz, 2H, ArH), 5.24 (d, J_{H4H3} 3.6 Hz, 1H, H4), 5.15 (dd, J_{H3H2} 10.3 J_{H3H4} 3.6 Hz, 1H, H3), 4.88 (dd, J_{H2H3} 10.3 J_{H2H3} 8.0 Hz, 1H, H2), 4.78 (d, J_{H1H2} 8.0 Hz, 1H, H1), 4.20 – 4.33 (m, 4H, H13, H12, H7), 4.16 (app. t, *J* 6.7 Hz, 1H, H5), 4.03 (d, J_{H6H5} 6.7 Hz, 2H, H6), 3.85 – 3.95 (m, 1H, H9), 2.09 (s, 3H, OCOC$\underline{H}_3$), 1.98 (s, 6H, OCOC$\underline{H}_3$), 1.92 (s, 3H, OCOC$\underline{H}_3$), 1.11 (d, J_{H8H7} 6.4 Hz, 3H, H8) ppm.

^{13}C NMR (101 MHz, DMSO-d_6) δ_C 170.40 (O$\underline{C}$OCH$_3$), 170.35 (O$\underline{C}$OCH$_3$), 170.0 (O$\underline{C}$OCH$_3$), 169.7 (O$\underline{C}$OCH$_3$), 156.5 (C11), 144.4 (Fmoc qC), 144.2 (Fmoc qC), 141.2 (2x Fmoc qC), 128.1 (Fmoc ArC), 127.5 (Fmoc ArC), 125.7 (Fmoc ArC), 120.6 (Fmoc ArC), 98.4 (C1), 75.4 (C7), 70.7 (C3), 70.1 (C5), 69.2 (C2), 67.6 (C4), 66.3 (C12), 61.3 (C6), 59.6 (C9), 47.1 (C13), 21.0 (OCO$\underline{C}$H$_3$), 20.93 (OCO$\underline{C}$H$_3$), 20.86 (OCO$\underline{C}$H$_3$), 20.8 (OCO$\underline{C}$H$_3$), 17.5 (C8) ppm.

HRMS (ESI$^+$) *m/z* calc. for $C_{33}H_{36}NO_{14}$ [M+H]$^+$ 670.2141; found 670.2144.

ν_{max}/cm^{-1} (neat) 2980 (ArC), 1748 (CO).

mp 123-125 °C.

General Procedures for Solid-Phase Peptide Synthesis (SPPS)

SPPS was performed manually in polypropylene syringe reaction vessels (10 mL; Torviq, MI, USA and 10 mL, 3 mL; Astech, Wicklow, Ireland) fitted with a polystyrene frit. All reactions were performed at room temperature under continuous agitation on an LBX Instruments Orb-B2 shaker set to 180 rpm. As all peptides synthesised contained Cys residues, the cleavage cocktail (TFA:EDT:H_2O:TES; 94:2.5:2.5:1 v/v) was exclusively used instead of the standard (TFA:TES:H_2O; 95:2.5:2.5 v/v) cocktail.

General Procedure 1: Resin Deprotection and Coupling of First Amino Acid (Rink Amide)

Rink amide AM resin (0.26 g, 0.2 mmol, 0.78 mmol/g, 100 - 200 mesh, 1.0 equiv.) was swollen with agitation in DMF (4 mL) in a SPPS syringe for 20 min and then drained. The resin-bound Fmoc protecting group was removed using 20% (v/v) piperidine in DMF (4 mL; 2 x 10 min) and the resin was washed with DMF (3 x 5 mL), DCM (3 x 5 mL) and DMF (3 x 5 mL). Fmoc-protected amino acid (4.0 eq.) was pre-activated with HATU (3.9 eq.) and DIPEA (8.0 eq.) in DMF (0.2 M) for 30 seconds and then transferred to the syringe containing the resin and agitated for 45 min. After this time, excess reagents were drained from the syringe and the resin was washed with DMF (3 x 5 mL), DCM (3 x 5 mL), DMF (3 x 5 mL). The coupling reaction and washes were then repeated.

General Procedure 2: Bromophenol Blue (BPB Monitoring of Coupling and Deprotection Reactions)

Successful coupling was qualitatively monitored by treatment of a small resin sample with a solution of bromophenol blue (BPB) in DCM (0.15 mM; 0.1 mL). For the monitoring of Fmoc-deprotection reactions, the beads would turn blue upon BPB treatment in the presence of a free amine; for the monitoring of amino acid coupling reactions, the beads would turn yellow. In the case of an incomplete deprotection or coupling, the associated procedure for the reaction was repeated.

General Procedure 3: Coupling of Subsequent Amino Acids

The subsequent amino acid coupling cycles consisted of **(i)** Fmoc deprotection with 20% (v/v) piperidine in DMF (4 mL; 2 x 10 min). **(ii)** Resin washes with DMF (3 x 5 mL), DCM (3 x 5 mL) and DMF (3 x 5 mL). **(iii)** Pre-activation of Fmoc-AA-OH (4.0 eq) with HATU (3.9 equiv.)/DIPEA (8.0 equiv.) in DMF (0.2 M) for 30 seconds followed by addition to the peptide resin and agitation for 45 min. **(iv)** (iii) was repeated for all amino acid couplings followed by washes with DMF (3 x 5 mL) between the repeat couplings. **(v)** Resin washes with DMF (3 x 5 mL), DCM (3 x 5 mL) and DMF (3 x 5 mL). **(vi)** Qualitative BPB test as per **General Procedure 2**. Following the final coupling, the resin was treated with 20% (v/v)

piperidine in DMF (5 mL, 2 x 10 min) and the resin was washed with 2 rounds of DMF (3 x 5 mL), DCM (3 x 5 mL), followed by drying of the resin under reduced pressure.

General Procedure 4: Coupling of Cysteine

Coupling of Fmoc-Cys(Trt)-OH/Fmoc-Cys(Acm)-OH consisted of **(i)** Fmoc deprotection by the addition of 20% (v/v) piperidine in DMF (4 mL) to the resin for 2 x 10 min, **(ii)** resin washes with DMF (3 x 5 mL), DCM (3 x 5 mL) and DMF (3 x 5 mL), **(iii)** peptide coupling with addition of DIC (4.0 equiv.), Oxyma Pure (4.0 equiv.) and Fmoc-Cys(Trt)-OH (4.0 equiv., 0.2 M) in DMF:DCM (1:1, v/v) were preactivated for 30 seconds and then added to the peptide resin for 45 min, **(iv)** resin washes with DMF (3 x 5 mL), DCM (3 x 5 mL) and DMF (3 x 5 mL), **(v)** qualitative BPB test as per **General Procedure 2**.

General Procedure 5: On-resin Deacetylation of Glycopeptides

Following a protocol reported by Galashov *et al.*,[6] in the final step prior to peptide cleavage, the resin was treated with a mixture of N_2H_4 (51% aq.)/DMF (1:1, v/v) for 30 minutes. Liquid was drained from the syringe and resin was washed DMF (3 x 5 mL), DCM (3 x 5 mL) and DMF (3 x 5 mL).

General Procedure 6: Global Deprotection and Resin Cleavage

The dried resin was swollen in DCM (4 mL) for 20 min, then drained and immersed in the cleavage cocktail (TFA:EDT:H_2O:TES; 94:2.5:2.5:1 v/v/v; 3 mL) unless otherwise stated, capped tightly and agitated for 90 min. The syringe was then drained, and the filtrate was collected. The resin was then washed with cleavage cocktail (3 x 2 mL) and the washings were combined with the initial filtrate. The combined filtrate was concentrated under a stream of N_2 to form an oily residue which was precipitated with ice-cold Et_2O (8 mL). The suspension was centrifuged and the supernatant was decanted. The pellet was resuspended in ice-cold diethyl ether (10 mL), centrifuged and the supernatant was decanted twice. After the final wash, the pellet was dried under a stream of N_2 and the crude peptide dried under reduced pressure.

Synthesis of Peptides

H_2N-Ala-Gly-Cys-Lys-Asn-Phe-Phe-Trp-Lys-Thr-Phe-Thr(β-Gal)-Ser-Cys-$CONH_2$ (S1)

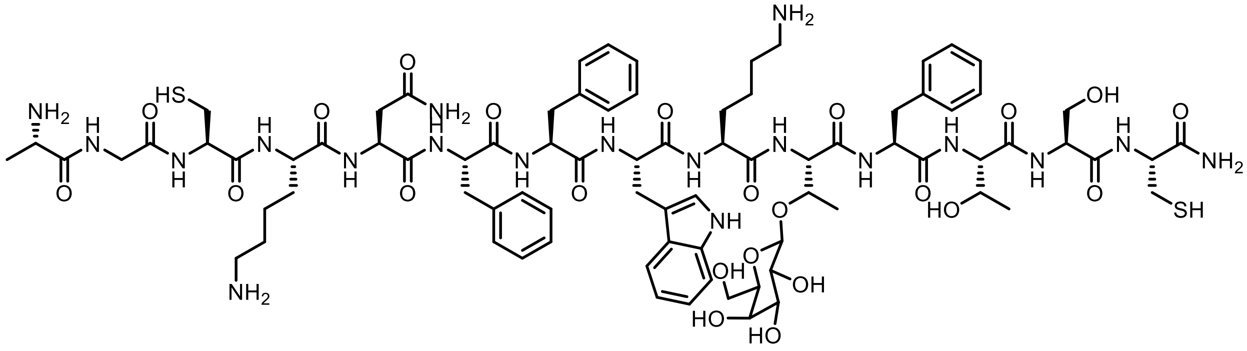

S1

Fmoc-Cys(Trt)-OH (586 mg, 1.00 mmol) was loaded to Rink Amide AM resin (321 mg, 0.25 mmol) as per a modified **General Procedure 1** using **General Procedure 4** for Cys coupling. Fmoc-Ser(^{t}Bu)-OH (383 mg, 1.00 mmol), Fmoc-Thr(^{t}Bu)-OH (397 mg, 1.00 mmol), Fmoc-Phe-OH (387 mg, 1.00 mmol), glycoamino acid **1** (671 mg, 1.00 mmol), Fmoc-Lys(Boc)-OH (469 mg, 1.00 mmol), Fmoc-Trp(Boc)-OH (527 mg, 1.00 mmol), Fmoc-Phe-OH (387 mg, 1.00 mmol), Fmoc-Phe-OH (387 mg, 1.00 mmol), Fmoc-Asn(Trt)-OH (597 mg, 1.00 mmol), Fmoc-Lys(Boc)-OH (469 mg, 1.00 mmol), Fmoc-Cys(Trt)-OH (586 mg, 1.00 mmol), Fmoc-Gly-OH (297 mg, 1.00 mmol), Fmoc-Ala-OH (370 mg, 1.00 mmol) were coupled according to **General Procedures 3** and **4**. The resin was split and a portion of the resin (0.1 mmol) was then treated with 20% (v/v) piperidine in DMF (5 mL; 2 x 10 min) followed by on-resin deacetylation according to **General Procedure 5** using N_2H_4 prior to cleavage. The peptide was cleaved from the resin using **General Procedure 6** yielding linear peptide **S1** (54 mg, 0.030 mmol, 30%) as a white fluffy solid.

HRMS (ESI$^+$) m/z calc. for $C_{82}H_{119}N_{19}O_{23}S_2$ $[M+2H]^{2+}$ 900.9078; found 900.9100.

R_T = 9.34 min (5 – 95% MeCN in H_2O with 0.1% TFA over 12 min, 220 nm).

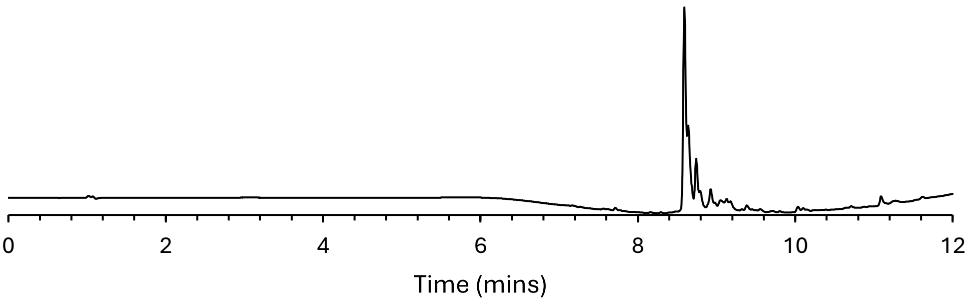

H_2N-cyclo(1,12)[Ala-Gly-Cys-Lys-Asn-Phe-Phe-Trp-Lys-Thr-Phe-Thr(β-Gal)-Ser-Cys]-$CONH_2$ (9)

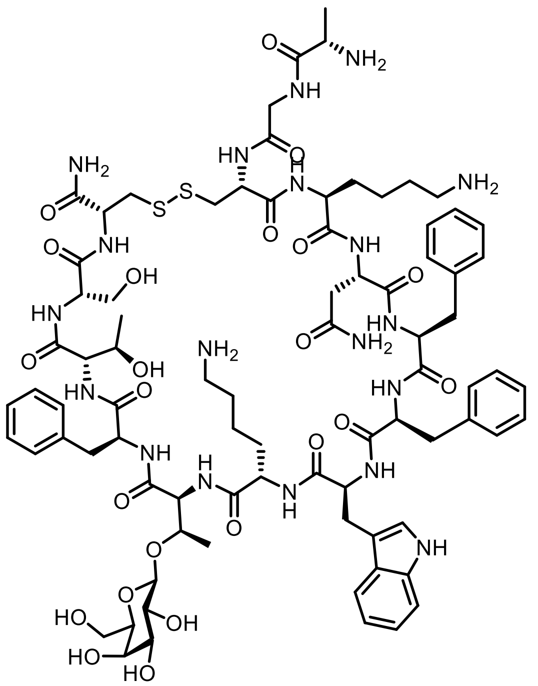

9

DMSO (0.5 mL) was added to a solution of linear peptide **S1** (10 mg, 5.5 µmol) in pH 7.4 phosphate buffer:MeCN (1:1 v/v; 4.5 mL) and stirred at rt for 48 h.. After this time, HPLC analysis showed consumption of the starting material (R_T = 9.34 min) and the formation of a new peak (R_T = 8.68 min). The crude material was purified by semi-preparative HPLC to give cyclic peptide **9** (1.1 mg, 0.60 µmol, 11%) as a fully white solid.

HRMS (ESI^+) *m/z* calc. for $C_{82}H_{117}N_{19}O_{23}S_2$ $[M+2H]^{2+}$ 899.9000; found 899.9018.

R_T = 8.68 min (5 – 95% MeCN in H_2O with 0.1% TFA over 12 min, 220 nm).

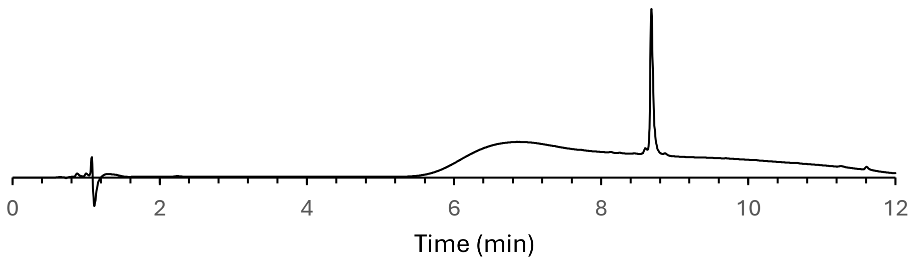

H_2N-Ala-Gly-Cys-Lys-Asn-Phe-Phe-Trp-Lys-Thr-Phe-Thr-Ser-Cys-$CONH_2$ (S2)

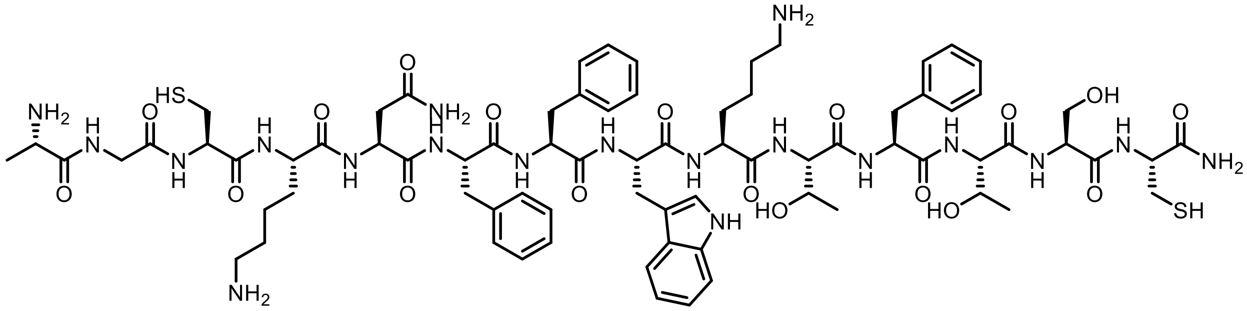

S2

Fmoc-Cys(Trt)-OH (586 mg, 1.00 mmol) was loaded to Rink Amide AM resin (321 mg, 0.25 mmol) as per a modified **General Procedure 1** using **General Procedure 4** for Cys coupling. Fmoc-Ser(^{t}Bu)-OH (383 mg, 1.00 mmol), Fmoc-Thr(^{t}Bu)-OH (397 mg, 1.00 mmol), Fmoc-Phe-OH (387 mg, 1.00 mmol), Fmoc-Thr(^{t}Bu)-OH (397 mg, 1.00 mmol), Fmoc-Lys(Boc)-OH (469 mg, 1.00 mmol), Fmoc-Trp(Boc)-OH (527 mg, 1.00 mmol), Fmoc-Phe-OH (387 mg, 1.00 mmol), Fmoc-Phe-OH (387 mg, 1.00 mmol), Fmoc-Asn(Trt)-OH (597 mg, 1.00 mmol), Fmoc-Lys(Boc)-OH (469 mg, 1.00 mmol), Fmoc-Cys(Trt)-OH (586 mg, 1.00 mmol), Fmoc-Gly-OH (297 mg, 1.00 mmol), Fmoc-Ala-OH (370 mg, 1.00 mmol) were coupled according to **General Procedures 3** and **4** . The resin was treated with 20% (v/v) piperidine in DMF (5 mL; 2 x 10 min) and the peptide was cleaved from the resin using **General Procedure 6** yielding linear peptide **S2** (249 mg, 0.15 mmol, 61%) as a white fluffy solid.

HRMS (ESI$^+$) *m/z* calc. for $C_{76}H_{109}N_{19}O_{18}S_2$ $[M+2H]^{2+}$ 819.8814; found 819.8835.

R_T = 9.14 min (5 – 95% MeCN in H_2O with 0.1% TFA over 12 min, 220 nm).

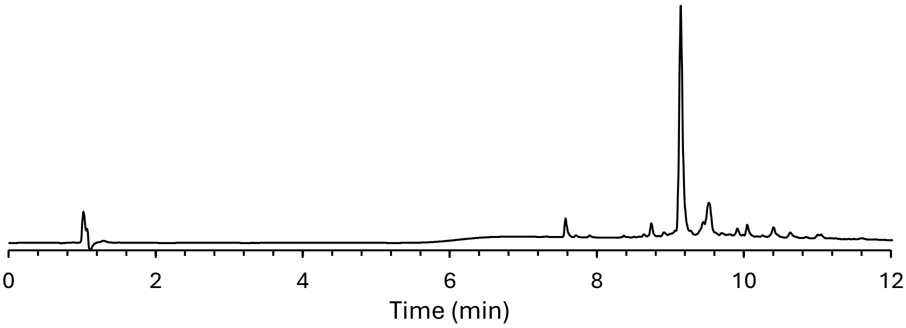

H_2N-cyclo(1,12)[Ala-Gly-Cys-Lys-Asn-Phe-Phe-Trp-Lys-Thr-Phe-Thr-Ser-Cys]-$CONH_2$ (10)

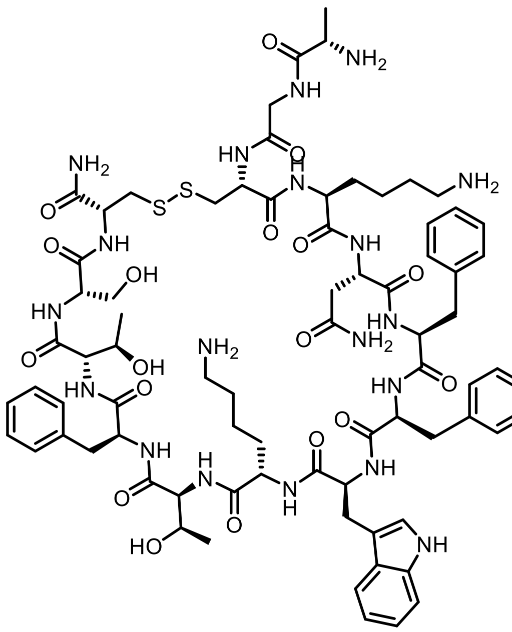

10

DMSO (1 mL) was added to a solution of linear peptide **S2** (20 mg, 12 µmol) in water:MeCN (1:1 v/v; 9 mL) and stirred at rt for 48 h. After this time, HPLC analysis showed consumption of the starting material (RT = 9.14 min) and the formation of a new peak (9.15 min). The crude material was purified by semi-preparative HPLC to give cyclic peptide **10** (1.7 mg, 1.04 µmol, 9%) as a white fluffy solid.

HRMS (ESI^+) *m/z* calc. for $C_{76}H_{107}N_{19}O_{18}S_2$ $[M+2H]^{2+}$ 818.8736; found 818.8748.

$\mathbf{R_T}$ = 9.15 min (5 – 95% MeCN in H_2O with 0.1% TFA over 12 min, 220 nm).

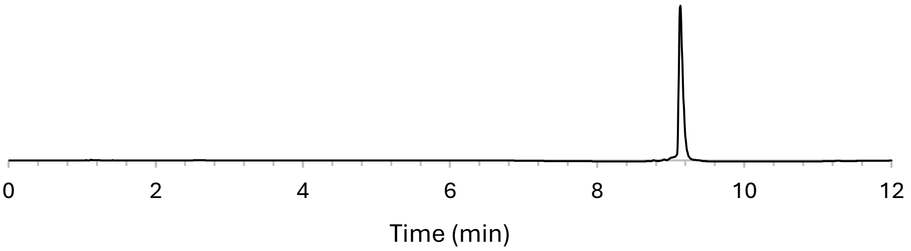

Biological Methods

MTT Assay

Human cervical carcinoma HeLa cells were maintained in DMEM – low glucose culture medium (D5921, Sigma-Aldrich), supplemented with 10% fetal bovine serum (FBS), 1% penicillin and streptomycin and 1% GlutaMAX^{TM} (35050-038, Gibco). Cells were grown in a normoxia condition (21% O_2), 5% CO_2 at 37°C. Once the cells reached 80%–90% confluency, they were dissociated into a single cell suspension with trypsin-EDTA at 37°C for 5 minutes and replated in T175 flasks at a density of 5,000 cells/cm^2.

The effects of the glycopeptide **9** and somatostatin-NH_2 **10** were evaluated using the MTT colorimetric assay, to determine their influence on cell metabolic activity.

Cells were plated at a density of 10,000 cells/well and in 96-wellplates (Nunc, Denmark) with 100 µL of culture medium. After 24 hours, the cells were subjected to various experimental conditions: glycopeptide 9 or somatostatin-NH_2 **10** alone (33 µM), glycopeptide **9** or somatostatin-NH_2 **10** in the presence of β-galactosidase (100 U/mL), and glycopeptide **9** or somatostatin-NH_2 **10** with both β-galactosidase (100 U/mL) and its relative inhibitor isopropyl β-D-1-thiogalactopyranoside (IPTG, 0.2 mM). Control (cell media only) and blank were included.

After 72 hours treatment, the cell culture media was carefully removed. Cells were washed twice with PBS before adding 100 µL of media and 20 µL of MTT solution (5 mg/ml in PBS) to each well. The plate was incubated in the dark at 37°C for 4 hours. After incubation, the supernatant was discarded, and the formazan crystals during the incubation period were dissolved by adding 120 µL of DMSO to each well. To ensure optimal crystal dissolution, the plate was shaken at 100 RPM for 15 minutes at 25°C. Then, supernatant was homogenised and 100 µL were transferred to a new clear flat bottom 96-wellplate for absorbance measurements.

Absorbance was measured at 570 nm using a plate reader (Synergy HT, BioTek). The absorbance reading from the blank control was subtracted from each absorbance measurement.

Each experiment was performed using five replicates (n=5) for each group. Data are presented as mean ± SD. Statistical analysis was performed using one-way ANOVA. Significance between different conditions in the same group is indicated as follows: $p < 0.0001$ (****). Significance compared to control is indicated as follows: $p < 0.01$ (##).

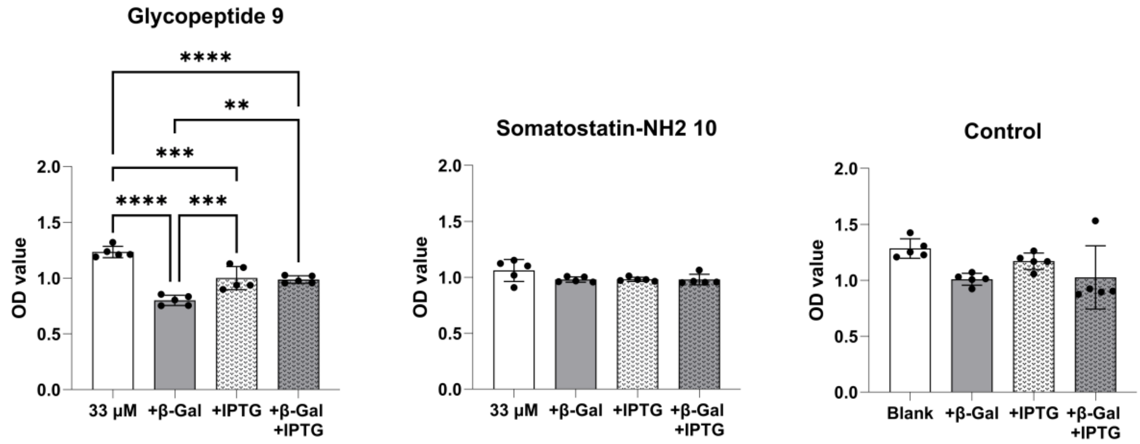

Figure S1 Comparison of glycopeptide **9**, somatostatin-NH_2 **10** and control (cell media only) responses in HeLa metabolic activity by MTT assay. HeLa metabolic activity was determined by MTT assay (n = 5) following 72 h treatment with glycopeptide **9**, somatostatin-NH_2 **10** (33 µM) or cell media only (control) under different conditions: i) glycopeptide **9** or somatostatin-NH_2 **10** (33 µM) alone or blank (control), ii) with 100 U/mL β-galactosidase (+β-Gal), iii) with 0.2 mM β-galactosidase inhibitor IPTG (+IPTG) iv) with 100 U/mL β-galactosidase and 0.2 mM β-galactosidase inhibitor IPTG (+β-Gal +IPTG). Results are presented as mean optical density (O.D.) ± standard deviation. Statistical analysis was performed using one-way ANOVA. Significance between different conditions in the same group is indicated as follows: $p < 0.0001$ (****). Significance compared to control is indicated as follows: $p < 0.01$ (##).

NMR Spectra

^{1}H NMR spectrum of **3** (400 MHz, $CDCl_3$)

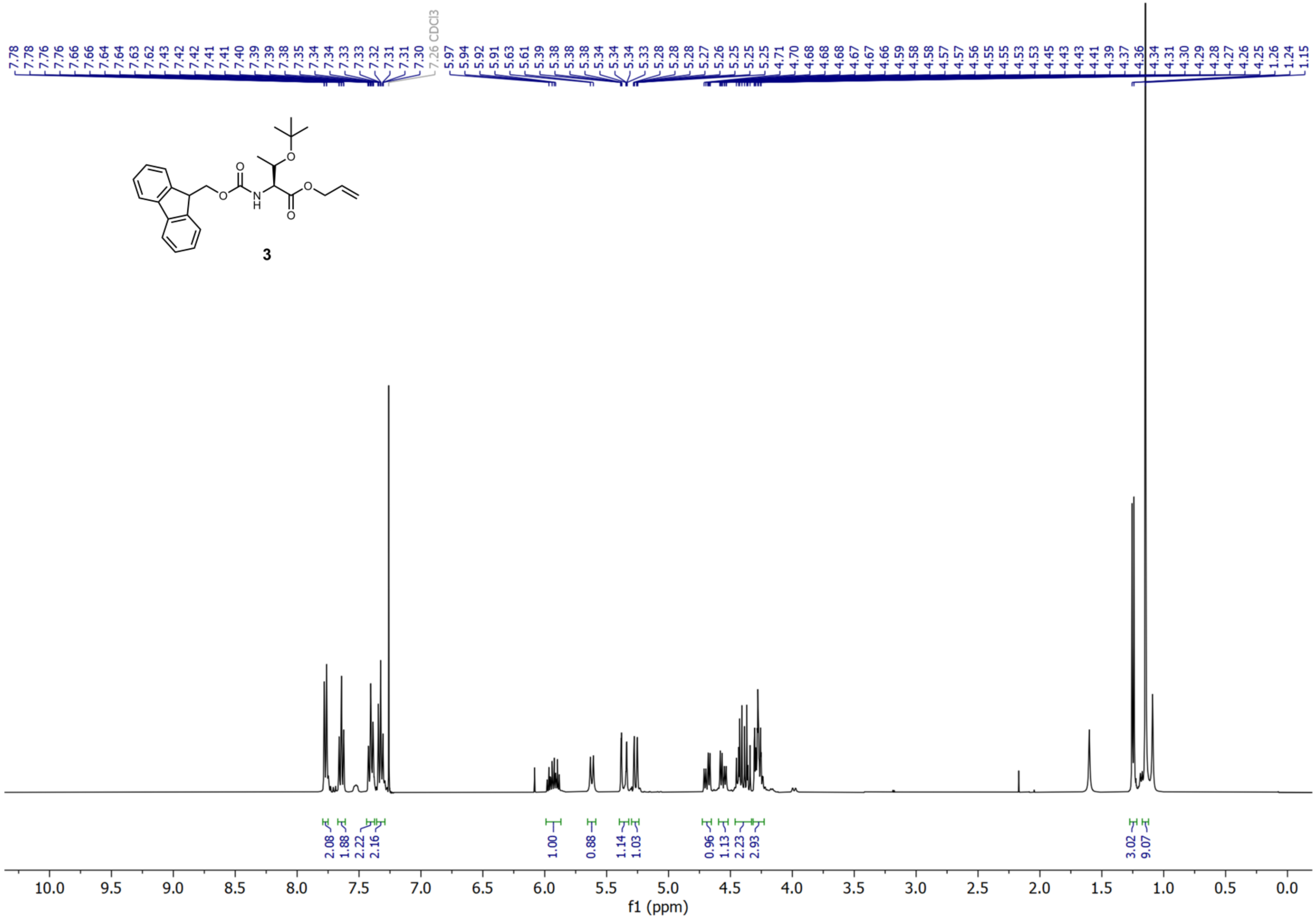

^{13}C NMR spectrum of **3** (101 MHz, $CDCl_3$)

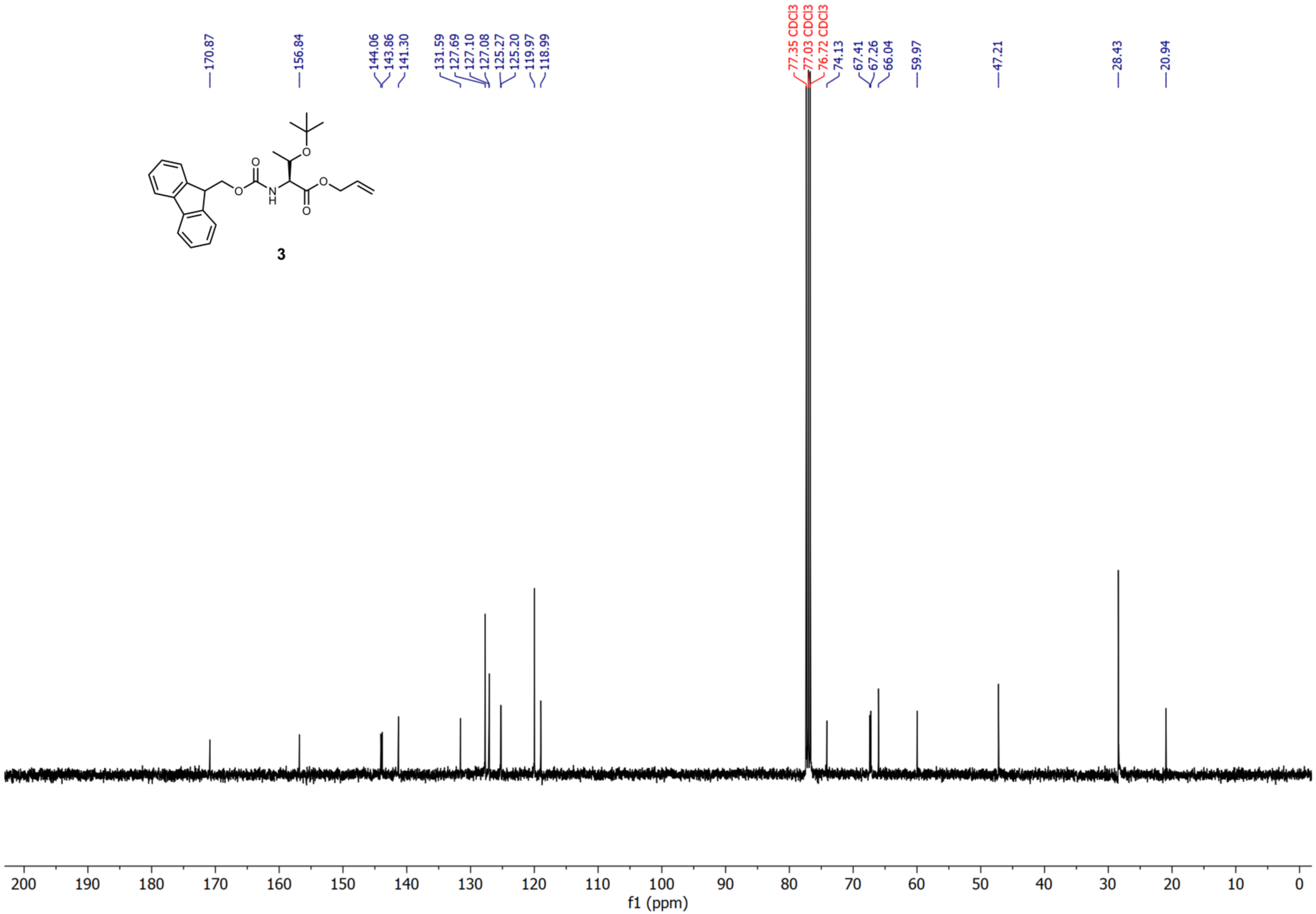

^{1}H NMR spectrum of **4** (400 MHz, DMSO-d$_6$)

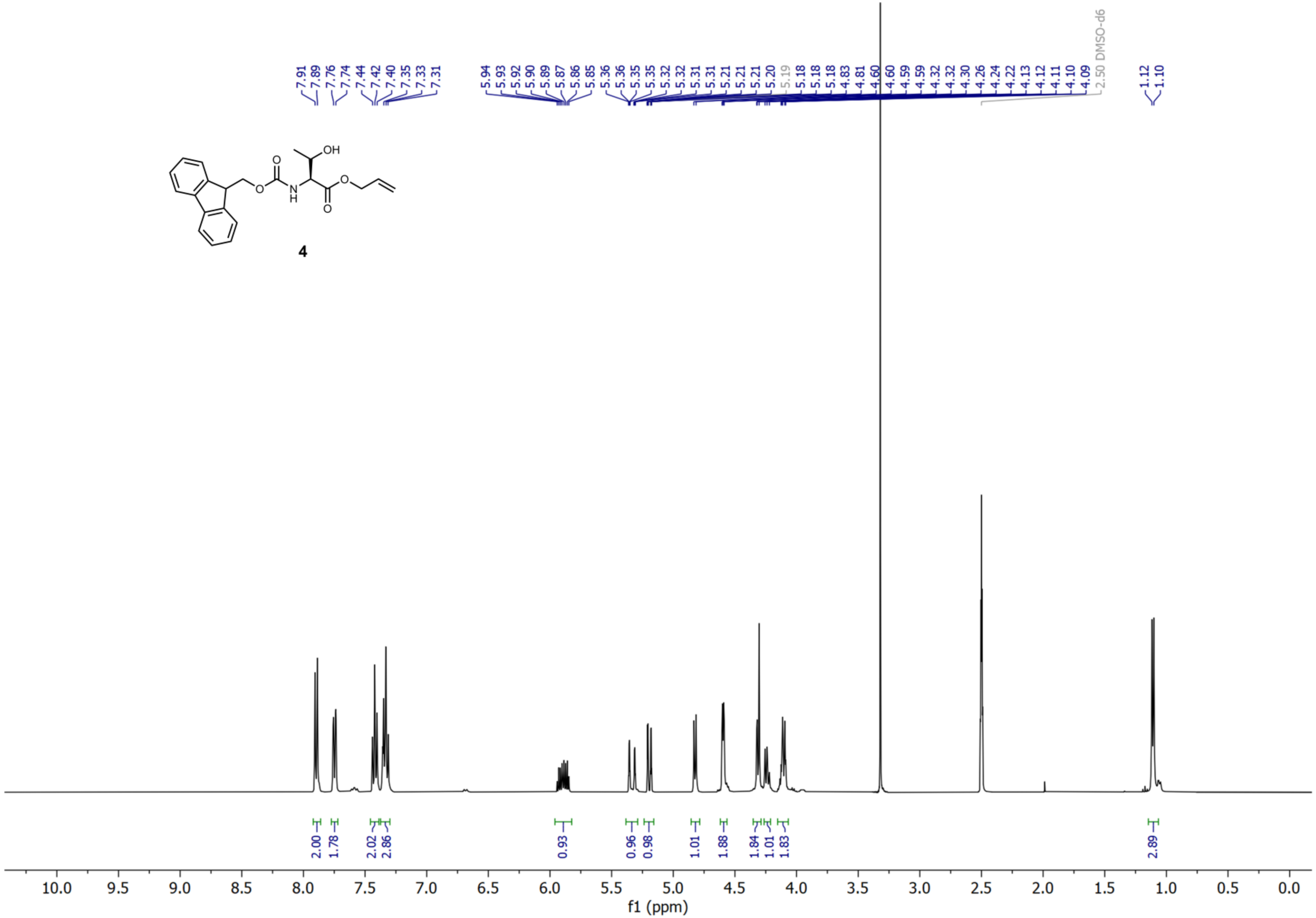

^{13}C NMR spectrum of **4** (101 MHz, DMSO-d$_6$)

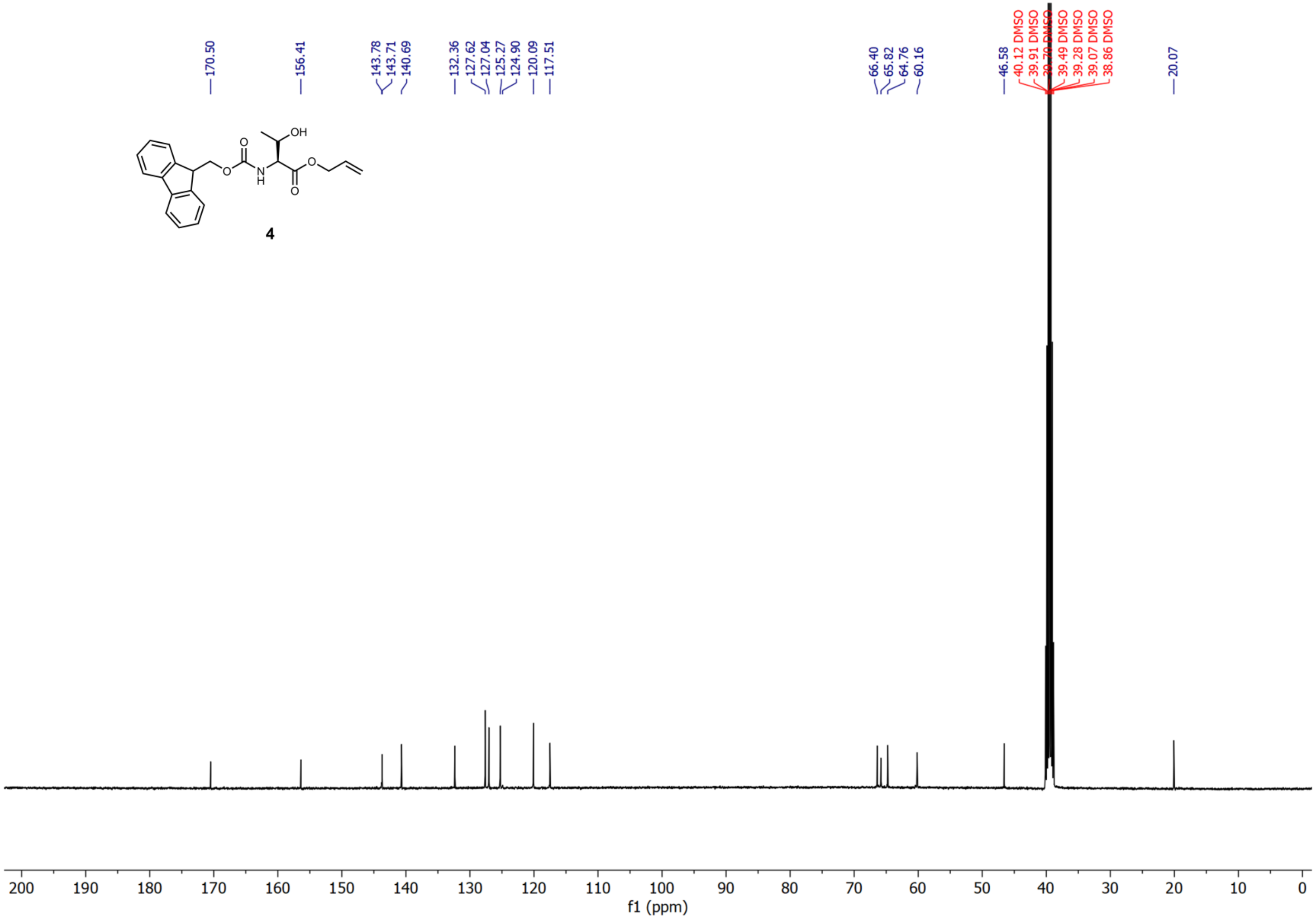

^{1}H NMR spectrum of **6** (400 MHz, $CDCl_3$)

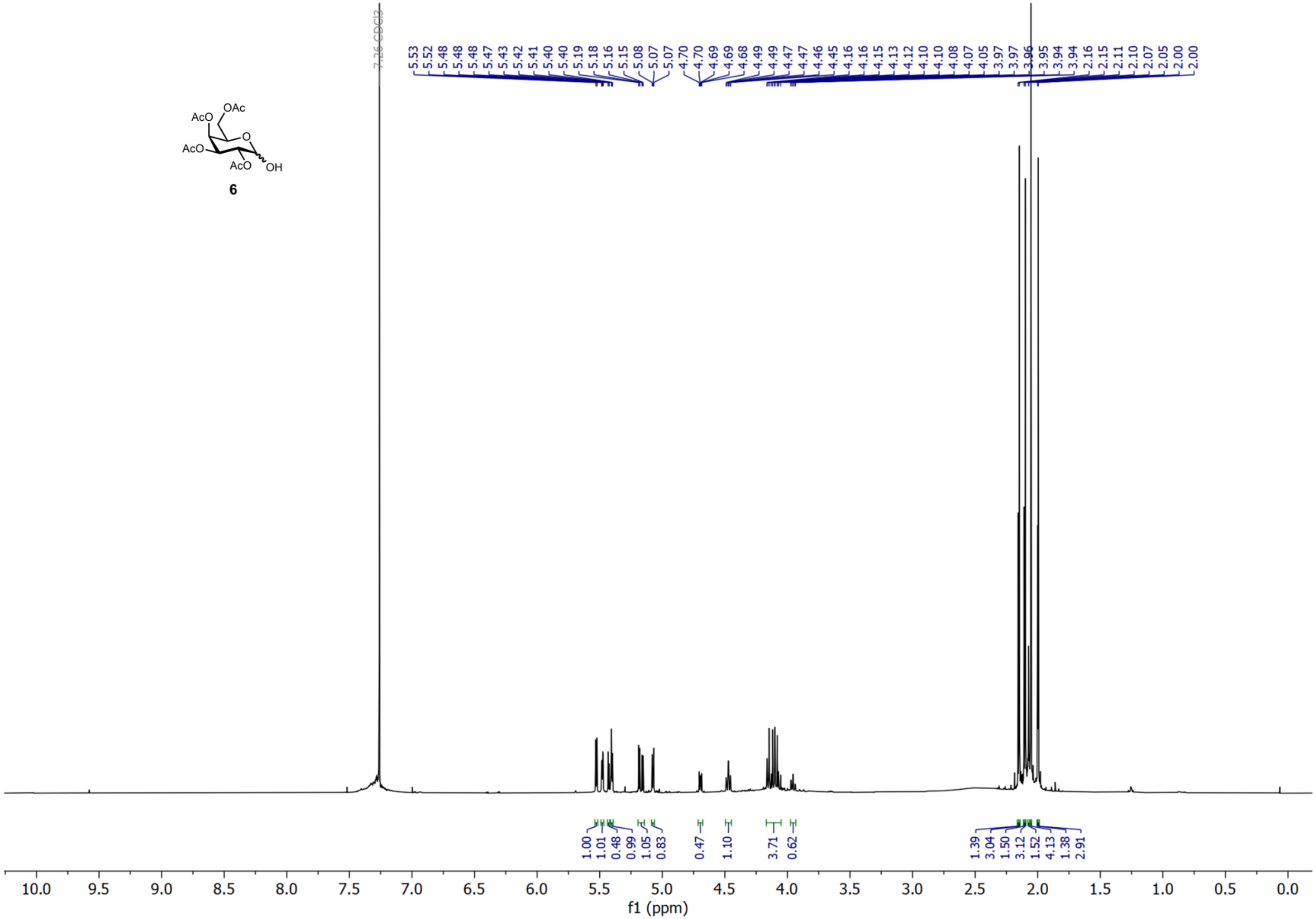

^{13}C NMR spectrum of **6** (101 MHz, $CDCl_3$)

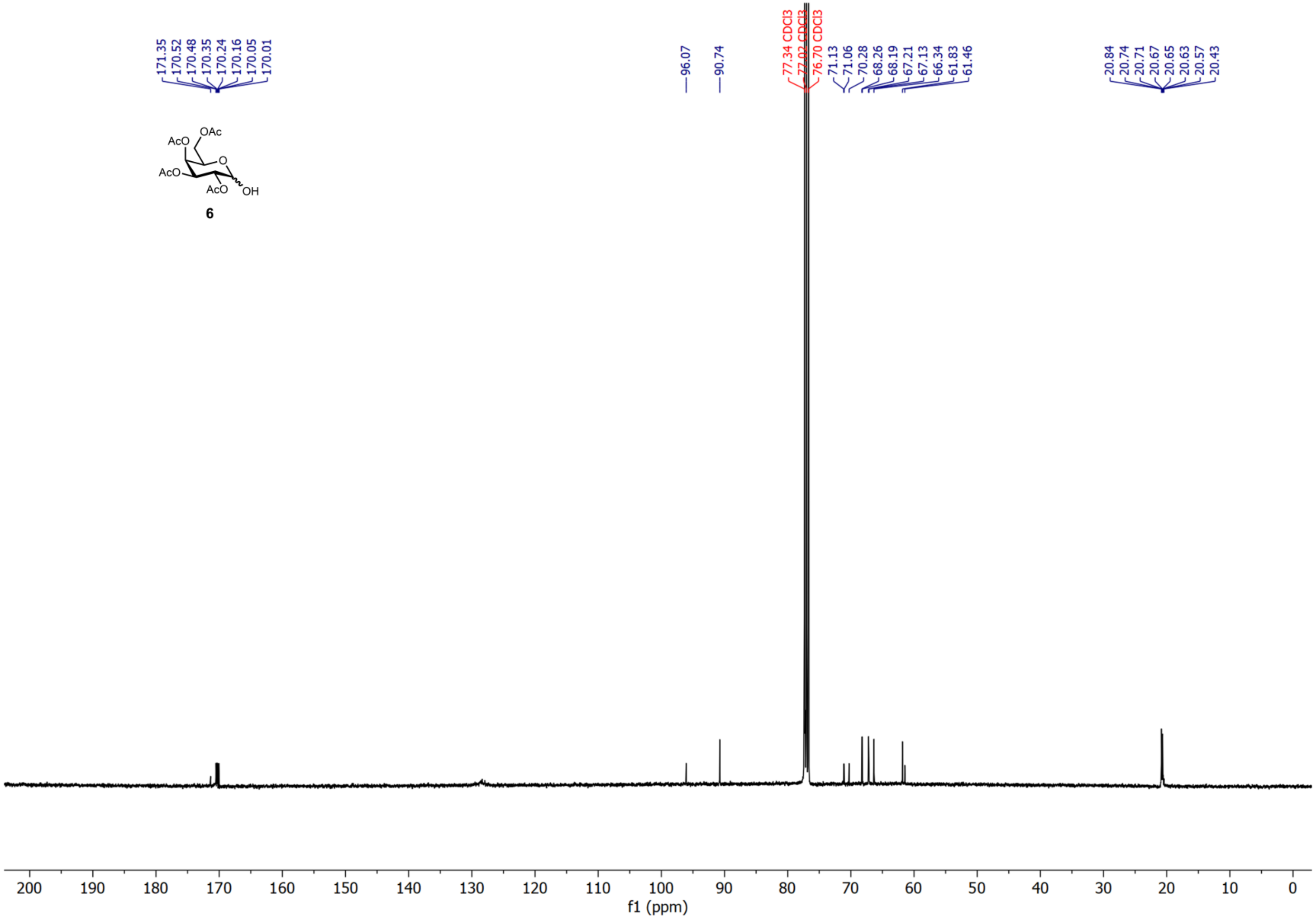

6

^1H NMR spectrum of **7 (α)** (400 MHz, $CDCl_3$)

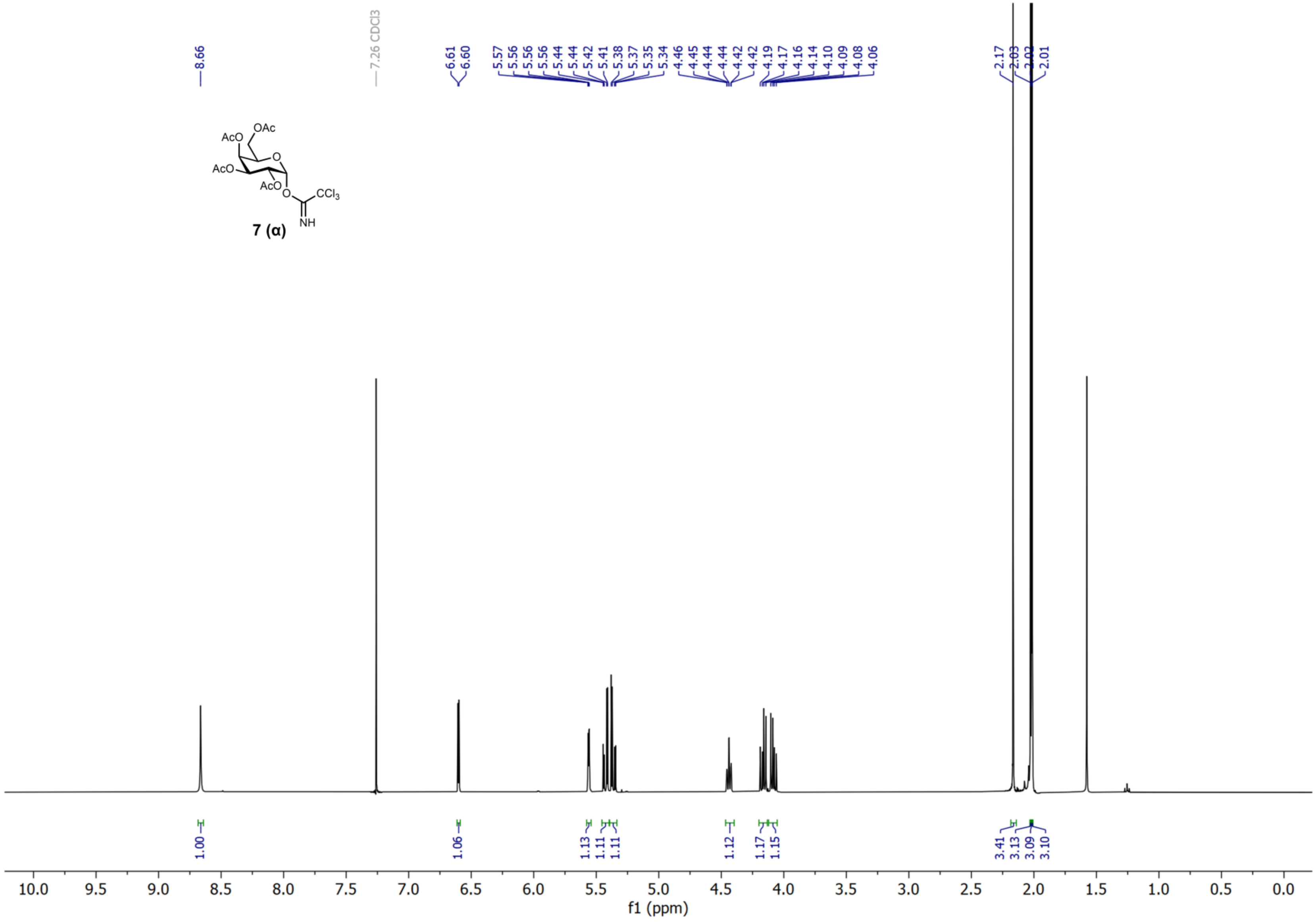

^{13}C NMR spectrum of **7 (α)** (101 MHz, $CDCl_3$)

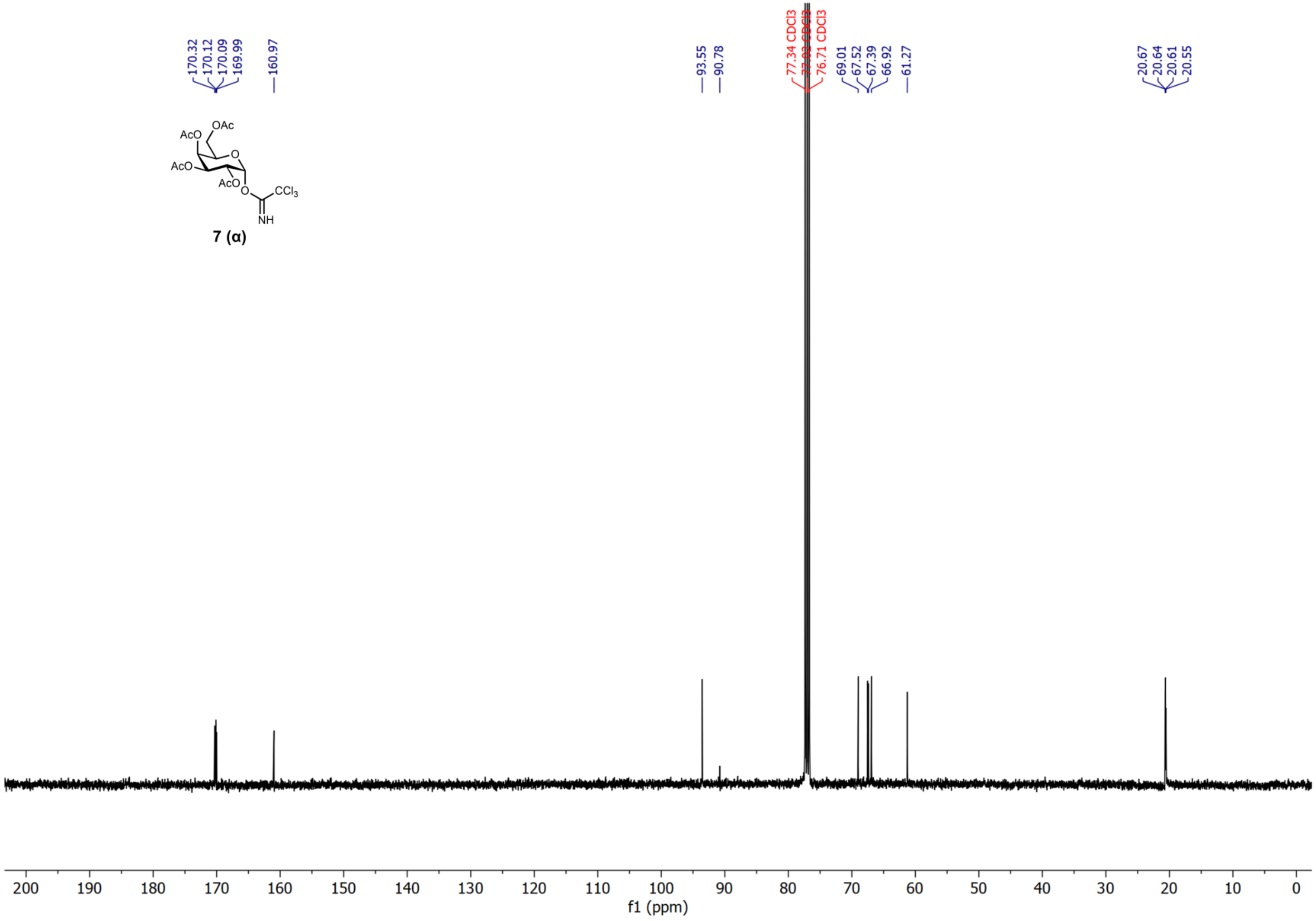

^1H NMR spectrum of **7 (β)** (400 MHz, $CDCl_3$)

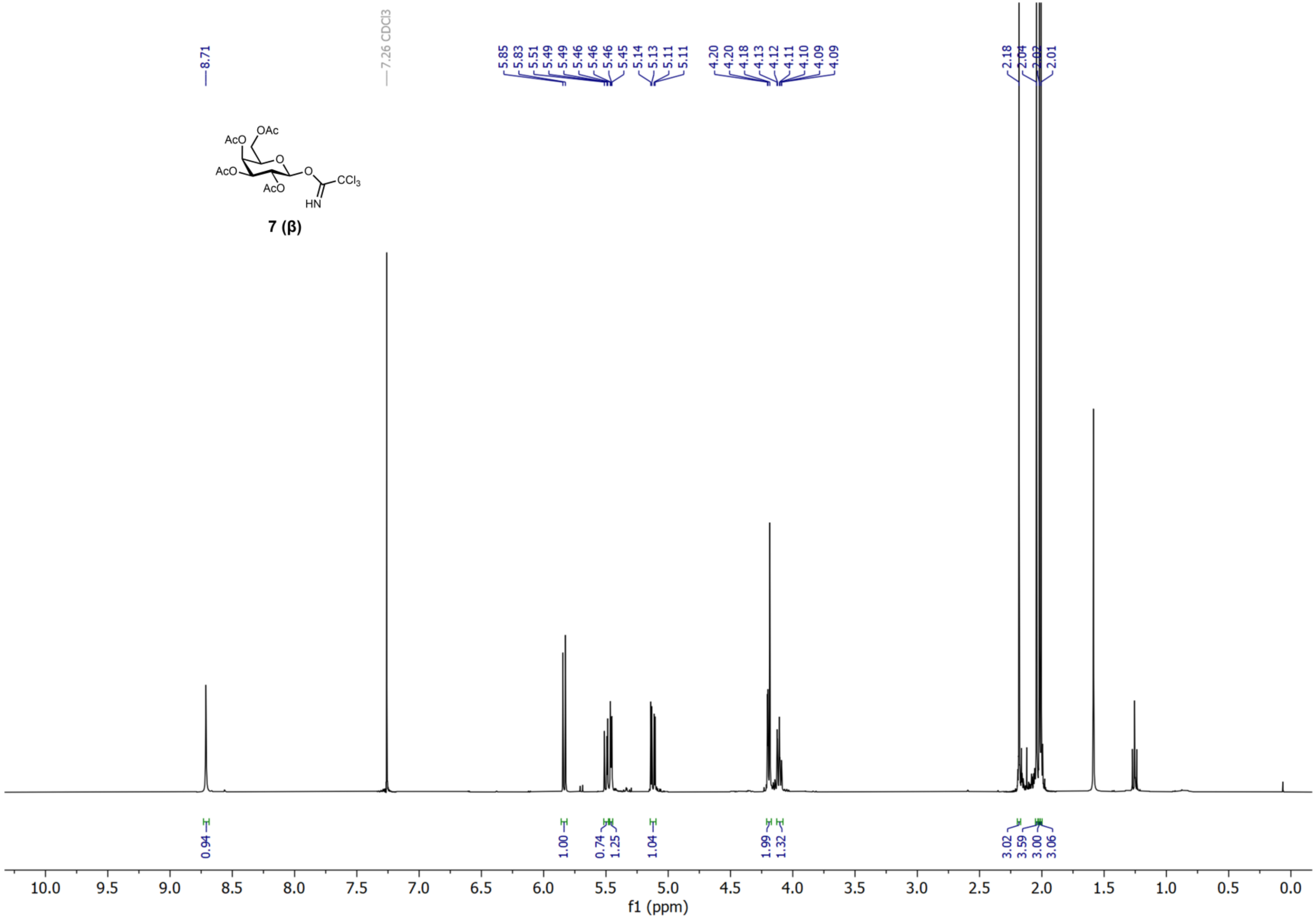

^{13}C NMR spectrum of **7 (β)** (101 MHz, $CDCl_3$)

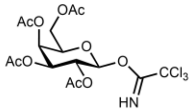

7 (β)

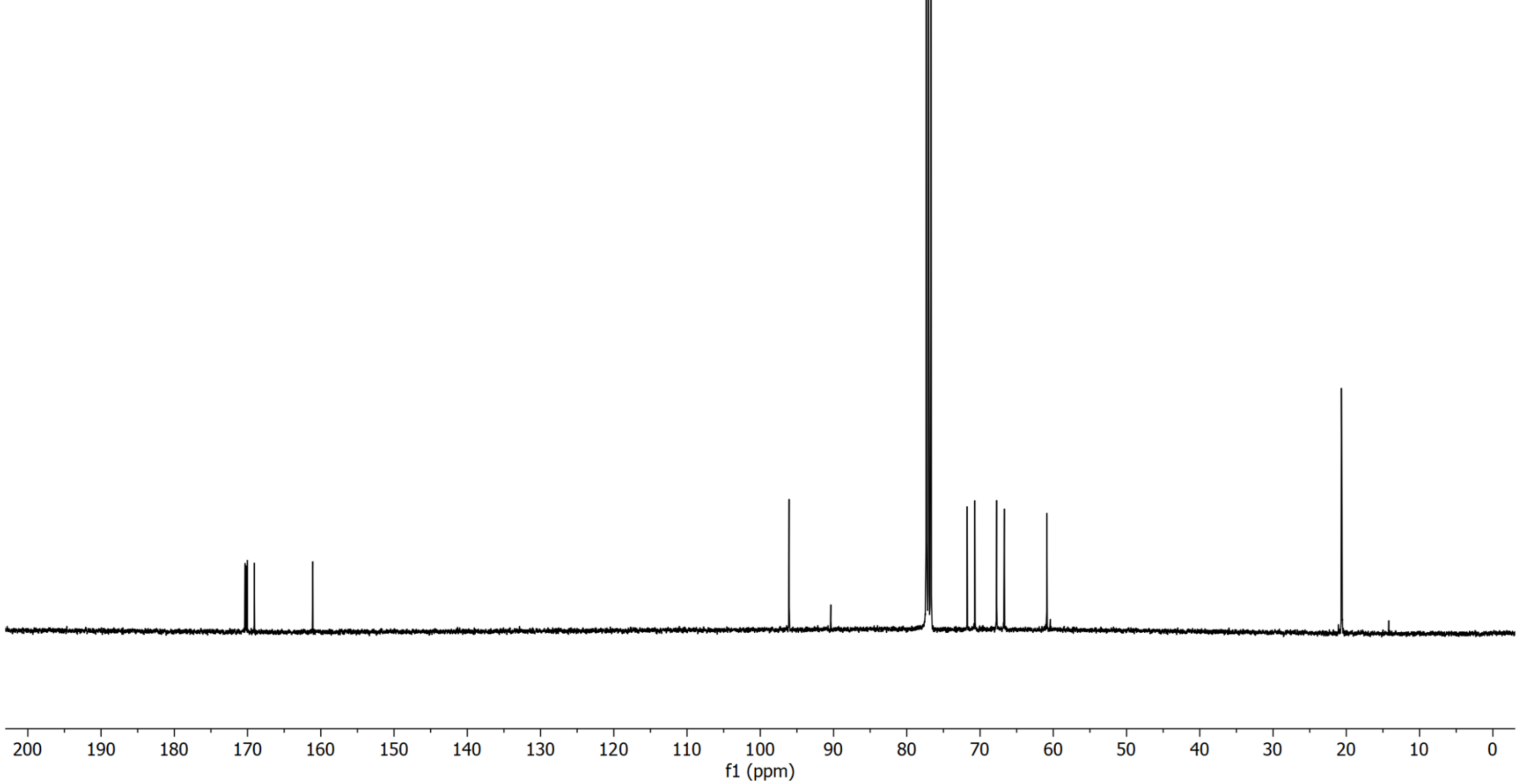

^{1}H NMR spectrum of **8** (400 MHz, $CDCl_3$)

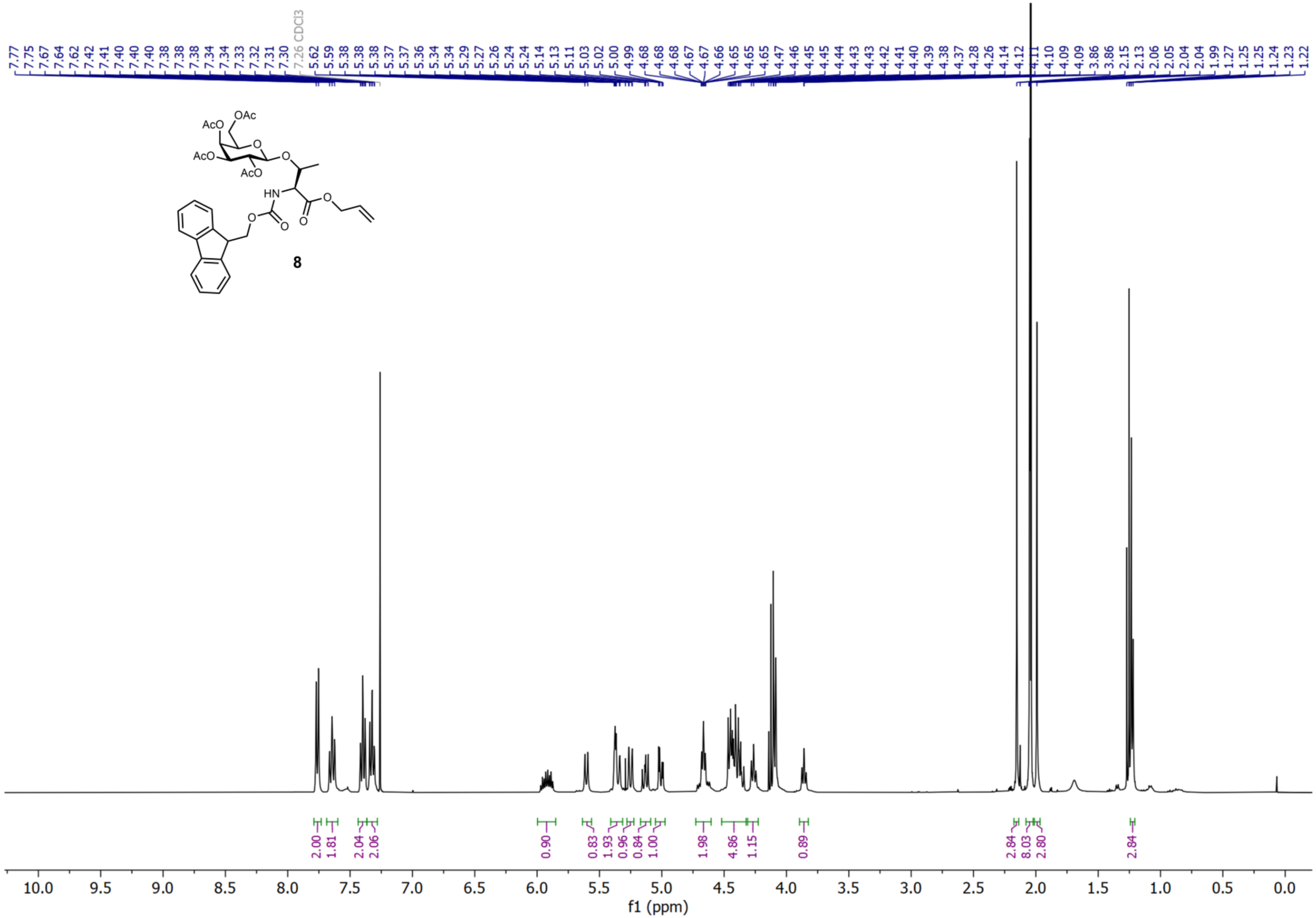

^{13}C NMR spectrum of **8** (101 MHz, $CDCl_3$)

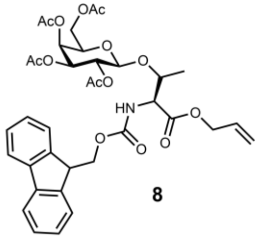

8

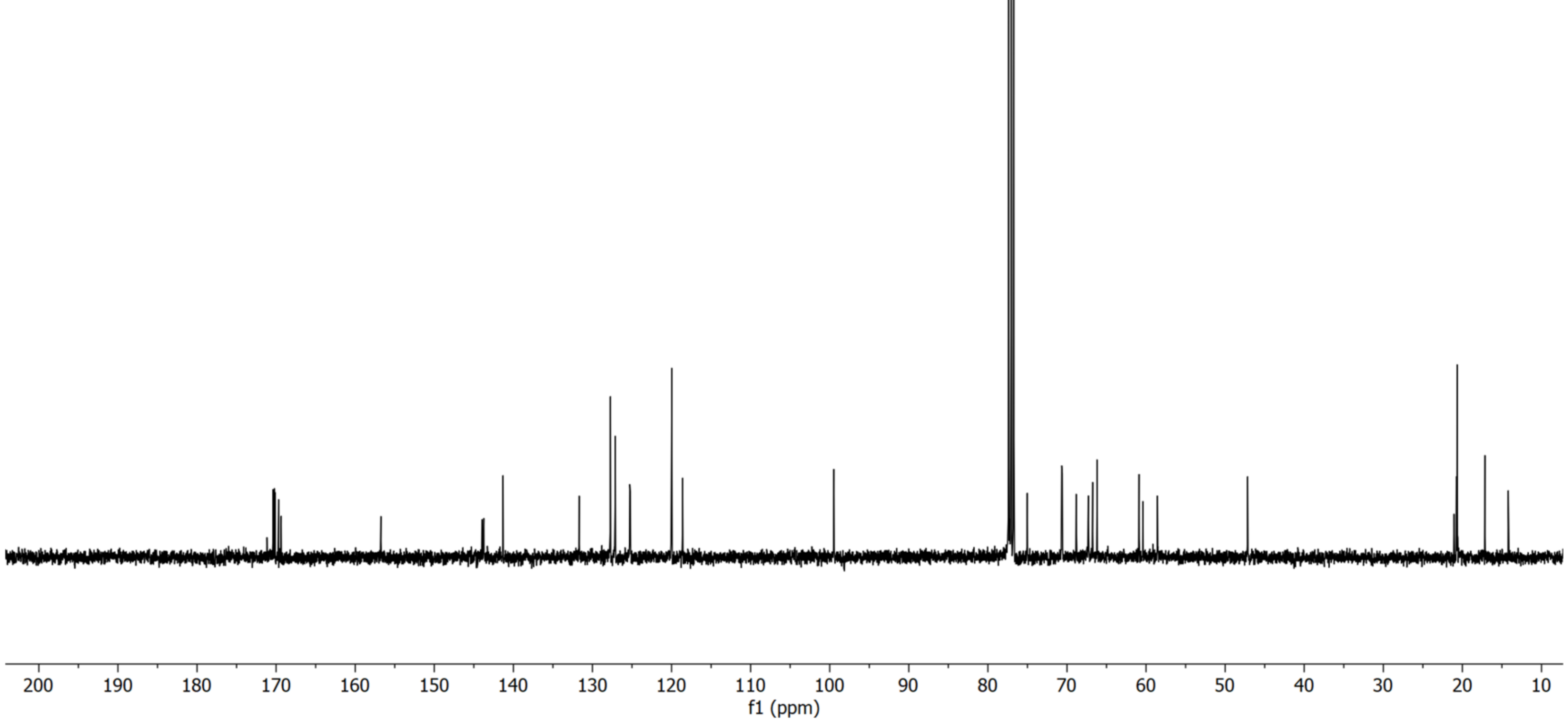

^{1}H NMR spectrum of **1** (400 MHz, DMSO-d_6)

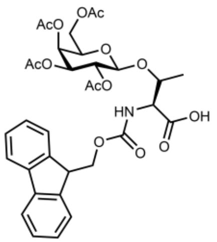

1

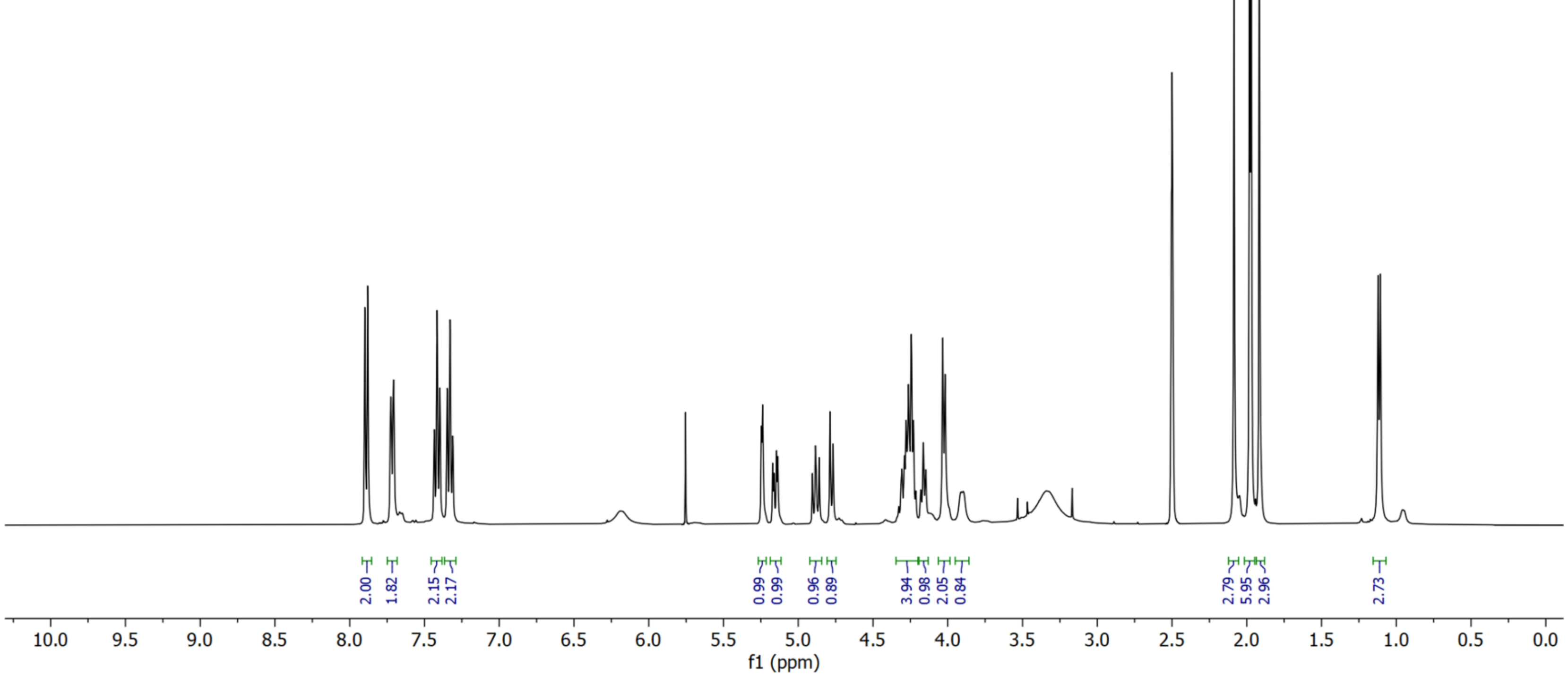

^{13}C NMR spectrum of **1** (101 MHz, DMSO-d$_6$)

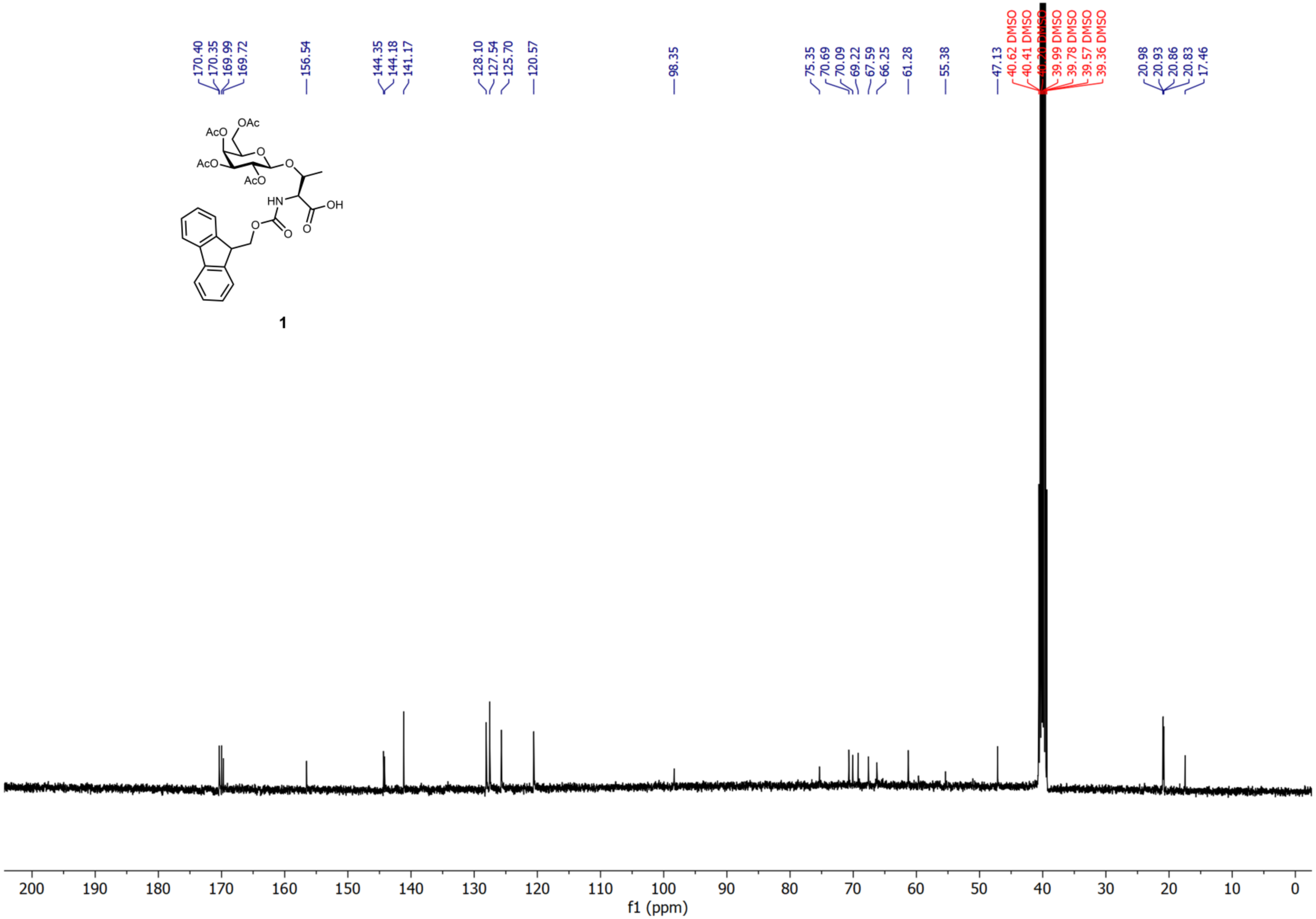

References

1. L. Rizzi, K. Cendic, N. Vaiana and S. Romeo, *Tetrahedron Letters*, 2011, **52**, 2808-2811.
2. T. B. Cai, D. Lu, X. Tang, Y. Zhang, M. Landerholm and P. G. Wang, *The Journal of Organic Chemistry*, 2005, **70**, 3518-3524.
3. P. H. Amvam-Zollo and P. Sinaÿ, *Carbohydr Res*, 1986, **150**, 199-212.
4. S. M. Andersen, M. Heuckendorff and H. H. Jensen, *Organic Letters*, 2015, **17**, 944-947.
5. R. Daly, G. Vaz, A. M. Davies, M. O. Senge and E. M. Scanlan, *Chemistry – A European Journal*, 2012, **18**, 14671-14679.
6. A. Galashov, E. Kazakova, C. E. Stieger, C. P. R. Hackenberger and O. Seitz, *Chemical Science*, 2024, **15**, 1297-1305.
